# Health and care service utilisation in the last year of life before non-sudden death in Wales, 2014–2023, by palliative care registration: a population-based retrospective cohort study

**DOI:** 10.1016/j.lanepe.2025.101479

**Published:** 2025-10-07

**Authors:** Rhiannon K. Owen, Rowena Bailey, Helen Daniels, Athena McBride, Ashley Akbari, Elinor Curnow, Alison Cooper, Natalie Joseph-Williams, Adrian Edwards, Maria Parry, Idris Baker

**Affiliations:** a Population Data Science, Swansea University Medical School, Faculty of Medicine, Health and Life Science, Swansea University, Swansea, UK; b Department of Population Health Sciences, Bristol Medical School, Medical Research Council Integrative Epidemiology Unit at the University of Bristol, University of Bristol, Bristol, UK; c Division of Population Medicine, Cardiff University, Cardiff, UK; d National Clinical Programme for Palliative & End of Life Care, Wales, UK

**Keywords:** Palliative care, End of life, Healthcare utilisation, Care home, Electronic health records, Routine data, Administrative data

## Abstract

**Background:**

End-of-life health and care service provision are complex processes. We aimed to quantify the uptake of health and care services in the last year of life before death from non-sudden causes by palliative care registration.

**Methods:**

Population-scale linked administrative and health data in the last year of life for Welsh residents who died of non-sudden causes were modelled using multi-state models between 2014 and 2023. Cox regression were used to estimate hazards for transitions between care settings, including people's homes, care homes with and without nursing, emergency, elective and other hospital admissions, and death. The primary outcome was rate of transition reported as hazard ratios (HR) with corresponding 95% confidence intervals (95% CI) adjusted for age, sex, rurality, area-level deprivation, and palliative care registration. Secondary outcomes included expected length of stay (ELOS).

**Findings:**

Our analyses included 267,199 individuals, with 1,845,572 transitions. There were 74,045 (27.7%) individuals registered for palliative care, under-represented groups included men, most-deprived and living alone. Most time was spent at home, with 370,752 (90.3%) of 410,441 emergency admissions from home. There was a 23% (HR 1.23 [95% CI 1.22–1.25]) increased transition rate of emergency admissions from home for palliative care registered compared with unregistered individuals, with a decreased expected length of stay (ELOS 25.34 [95% CI 25.34–25.34] vs 26.87 [26.87–26.87]). Emergency admissions from care homes with and without nursing were 17% (HR 0.83 [95% CI 0.80–0.86]) and 18% (HR 0.82 [95% CI 0.79–0.85]) lower for palliative care registered compared with unregistered individuals, with an increased rate of discharge from emergency hospital settings (HR 2.00 [95% CI 1.92–2.09] and 1.62 [1.54–1.69]).

**Interpretation:**

Palliative care status was associated with health and care utilisation at the end-of-life. Efficient identification of individuals needing palliative care and additional support at home should be prioritised for system optimisation.

**Funding:**

Health and Care Research Wales Evidence Centre.

**Research in context**

**Evidence before this study**

We searched PubMed and relevant citations on February 20, 2025 using the search terms ((health service utilisation [Title/Abstract]) OR (health care utilisation [Title/Abstract]) OR (pathway [Title/Abstract])) AND ((end of life [Title/Abstract]) OR (last year of life [Title/Abstract]) OR (palliative care [Title/Abstract])) AND ((administrative data) OR (health record) OR (health data) OR (administration data)), which returned 281 studies. These studies have focused on evaluating health and care service utilisation towards the end-of-life within specific settings (e.g., hospitals or hospice care) and/or targeted populations (e.g., paediatrics or cancer). Overall, these studies found that there was increased health and care service utilisation with increasing proximity to death. Whilst some of these studies are population-based, none of these studies comprehensively assessed utilisation across both health and care services, including care homes with and without nursing, on a population-scale for all individuals nearing the end-of-life who died from non-sudden causes. Existing studies also do not compare pathways of health and care service utilisation by palliative care registration.

**Added value of this study**

This study explores the patterns of change in both health and care settings at a system level using population-scale data in the last year of life for individuals who died of non-sudden causes. In this study, we account for and assess competing transitions to health and care services at an individual level (e.g., for an individual living at home, transition to emergency hospital admission is a competing pathway for admission to care homes), and explore characteristics associated with uptake. The findings of this study could be used to inform healthcare policy and practice to provide optimised care at the end-of-life in a population with access to universal healthcare.

**Implications of all the available evidence**

As demand for end of life services increases with ageing populations, health and care services need to adapt to provide efficient and appropriate management and care. Existing evidence suggests that health and care service utilisation increases with closer proximity to death, and the economic impact of end-of-life care is substantial. This population-scale analysis found that demand for urgent care increased rapidly towards the end-of-life. People in urban areas and those in receipt of palliative care had an increased rate of health and care service utilisation from home compared to those from rural areas and not in receipt of palliative care, respectively. However, those on the palliative care register had a decreased rate of health service utilisation from care homes with and without nursing compared to those not on the palliative care register. Overall, individuals on the palliative care register were discharged from emergency hospital settings at a faster rate and therefore, had a reduced expected length of stay. Men, residents from urban areas, those living in the most-deprived communities and those living alone were under-represented on the palliative care register. Targeted approaches for efficiently identifying individuals needing palliative care services and additional support provided at home, where appropriate, should be prioritised to optimise system management and appropriate care for those nearing the end-of-life.

## Introduction

As global populations live longer, there are increasing numbers of people living with multiple long-term conditions, with increasing demand on health and care services, including palliative care services.^1^ It is estimated that between 75% and 90% of those nearing the end-of-life have a need for some form of palliative care,^2^^,^^3^ with a projected 160,000 more people in England and Wales requiring palliative care services by 2040.^4^ As a result, health and care services will need to adapt to meet the needs of evolving populations.

Engagement with health and care services at the end-of-life reflects complex systems.^5^ Existing evidence focuses on assessing healthcare utilisation among individuals at the end-of-life or in receipt of palliative care within specific settings such as emergency department attendances or hospital admissions,^6^, ^7^, ^8^, ^9^, ^10^, ^11^ outpatient palliative care,^12^^,^^13^ or hospice care.^14^ Other studies have explored healthcare utilisation patterns using summary statistics.^15^^,^^16^ A further body of work has evaluated specific populations such as paediatric palliative care,^6^ chronic obstructive pulmonary disease,^13^ cancer,^17^ heart failure,^9^ chronic kidney disease,^11^ and multiple long-term conditions.^10^ Other studies have aimed to predict mortality in individuals nearing the end-of-life to identify those who may benefit from earlier receipt of palliative care services.^18^ These studies have been undertaken in a range of countries, including Australia,^6^^,^^11^ United States of America (USA),^7^^,^^10^^,^^12^^,^^14^^,^^18^ Canada,^9^^,^^13^ and several European countries.^8^^,^^15^, ^16^, ^17^

Little is known about health and care service utilisation at a system level for those nearing the end-of-life in a United Kingdom (UK) population, with access to universal healthcare. Previous studies in the UK have summarised healthcare utilisation^19^ and place of death^20^ using summary statistics. Other studies have compared healthcare utilisation using formal statistical testing for individuals in the last year of life,^21^ and specifically for people dying of cancer.^22^^,^^23^ A number of studies have evaluated the economic impact of those in the last year of life,^21^ including an evaluation of public expenditure across both health and social care settings.^24^ The objective of this work was to assess how individuals interact with both health and care services in the last year of life, accounting for competing pathways of care at a system-level, and examine whether the rate of health and care service utilisation by setting and type of care differs for those in receipt of palliative care and by demographic characteristics. More generally, our work provides a population-scale time-resolved analysis to provide information on health and care service pathways in the last year of life for individuals with access to universal healthcare, which has the potential to inform healthcare service design and delivery in the UK and beyond.

## Methods

### Study design and setting

We conducted a population-scale retrospective cohort study using data held within the Secure Anonymised Information Linkage (SAIL) Databank between 1st January 2014 and 31st December 2023.^25^ The SAIL Databank contains anonymised individual-level population-scale demographic, mortality, and electronic health record (EHR) data for the resident population of Wales or those in receipt of NHS Wales services. The SAIL Databank independent Information Governance Review Panel (IGRP) approved this research under SAIL Project 1641 including the pre-planned analyses.

### Data sources

Demographic, mortality, and electronic health record (EHR) data sources were linked using anonymised linkage fields (ALFs) at the individual-level with complete coverage, meaning all admissions were captured for all individuals.^26^ Demographic data, including anonymised residential addresses, were obtained from the Welsh Demographic Service Dataset (WDSD), which holds administrative information for the population of Wales that are registered to a Welsh General Practice. Anonymised residential addresses were recorded using a residential anonymised linkage field (RALFs), which are mapped to a corresponding Health Board, Local Authority Lower-layer Super Output Area (LSOA), and Office for National Statistics (ONS) rural-urban classification,^27^ and were used to identify individuals living alone at cohort start. Area-level deprivation was described using the Welsh Index of Multiple Deprivation (WIMD; version 2019),^28^ which was calculated for each LSOA excluding the health domain (since it includes death). These were ranked and subsequently divided into fifths to create quintiles from 1 to 5, where 1 represents ‘the most deprived’ and 5 ‘the least deprived’ LSOAs.

The Care Home data source (CARE) contains a record of anonymised care homes in Wales registered with the Care Inspectorate Wales (CIW). These data were linked to residential addresses using the RALF to identify care home residencies at any point in time (including temporary stays). The CARE data were used to identify the type of care home, including nursing and non-nursing care. Mortality data were obtained from the Annual District Death Extract (ADDE) from the ONS mortality register, which holds information regarding the dates and cause of death (recorded as International Classification of Diseases version 10 (ICD-10) codes) for all Welsh residents (including those who died outside of Wales). Emergency department admissions were identified using the Emergency Department Dataset (EDDS), which contains information for all NHS Wales Accident and Emergency department attendances. The Patient Episode Database for Wales (PEDW) contains all secondary care data in Wales and was used to capture elective and other admissions. The Welsh Longitudinal General Practice (WLGP) data were used to identify individuals registered in receipt of palliative care using Read version 2 codes (provided in Supplementary Table S1) and to calculate the electronic frailty index (eFI) using a 10-year look back window from the date of cohort entry. The eFI scores were categorised as: fit (eFI value of 0–0.12), mild (>0.12–0.24), moderate (>0.24–0.36) or severe frailty (>0.36)^29^ or missing. The WLGP records attendance and clinical information for all primary care interactions, including patient symptoms, investigations, test results, diagnoses, prescribed medication, and referrals to tertiary care.

### Participants

We identified individuals who had died of non-sudden causes between 1st January 2015 and 31st December 2023, defined as deaths excluding external causes of morbidity and mortality using ICD-10 chapters 19 and 20, which is similar to the maximal estimate approach described by Rosenwax et al.,^30^ with the exception of including conditions originating in the perinatal and pregnancy periods to capture all individuals who may be potentially eligible for palliative care services. The cohort was restricted to those with at least one known residency in Wales during the last year of life to ensure that we did not exclude anyone in a long-term healthcare setting (who therefore may not have a residential address) at cohort start. The start of follow-up was one year prior to their date of death, which included follow-up from 1st January 2014 to 31st December 2023. Only individuals with complete follow-up were included in the analysis, meaning that individuals who migrated in or out of Wales during the last year of life were excluded from the cohort (n = 17,530, 6%) to ensure that complete care pathways were captured.

### Outcome measures

The primary outcome was the rate of transition between home (defined as an individual's own home), nursing care home, care home without nursing, elective admission, emergency admission or other hospital admission (including babies born within the healthcare provider, transfer of admitted patients from other settings, and transfer to General Practice care in community hospitals)^31^ for those on the palliative care register compared with those not on the palliative care register. Palliative care registered individuals were identified from the WLGP data source using Read version 2 codes (Supplementary Table S1) that are used by the Quality Outcomes Framework with additional codes recommended by the Gold Standard Framework,^32^ and includes palliative care received from all healthcare professionals. Registration entails a (GP) practice-based list of patients with palliative care needs, of varying severity or urgency, kept regularly under review and in discussion with local palliative care team staff. Results were reported as adjusted hazard ratios and associated 95% confidence intervals, and illustrated using cumulative incidence plots. Secondary outcome measures included frequency of health state transitions and expected length of stay (ELOS) restricted to 1-year, for each transition. ELOS was used as it correctly accounts for the time spent in each state across all possible transition paths and censoring patterns.^33^ States in the care pathway were defined using the date of residential address, admission and discharge to care and/or hospital, and date of death. We were unable to capture information on stays in hospices, meaning that periods of time spent in a hospice were treated as time at home unless recorded as a change of address or linked to a residential care home.

### Statistical analysis

Multi-state models^34^ were used to model trajectories of care pathways, accounting for competing pathways including death. The similarity between transitions within an individual were accounted for using robust standard errors.^35^ Supplementary Figure S1 illustrates the modelling framework. Individuals can start at home, or in a care home without nursing, care home with nursing, emergency, elective or other admission at cohort inception. Individuals could forward and backward transition to all states except death, where death was included as an absorbing state. Transition to death from any state is not interchangeable with place of death. For example, transition from home to death does not necessarily mean the individual died at home—it is possible that individuals died in an ambulance or hospital (e.g., emergency department before being formally admitted) or in a hospice setting. The model allows simultaneous estimation of all health and care pathways via estimation of all possible transitions between states at every time point. Time to transition or time to death in days was used as the timescale. Exact times of day were used when individuals transitioned to multiple states on the same day, such that days were measured to the nearest two decimal places. Separate baseline hazards were assumed for each of the transitions. Cox Proportional Hazard regression models were used to adjust for covariate effects. All analyses were adjusted for age at cohort start, sex, area-level deprivation using the Welsh Index of Multiple Deprivation (WIMD) version 2019 quintiles, rurality, and palliative care registration. We aimed to compare transition rates by palliative care registration and rurality. For computational stability, age, sex and area-level deprivation were assumed to have a common effect across transitions. Rurality and palliative care registration were assumed to vary across transitions. The proportional hazards assumption was assessed using Schoenfeld residuals. We report hazard ratios with associated 95% confidence intervals. Restricted expected length of stay (ELOS), restricted to 1-year, within each state is calculated using transition probabilities,^33^ with associated standard errors estimated using an extension of the methods of moments approach to the multi-state model setting.^36^ Results are illustrated using cumulative incidence plots based on a 75-year old, male, from the most deprived community, living in an urban setting. Results for younger ages (25 and 50 years), females, least deprived populations, and rural settings are provided in the Supplementary Materials. Sensitivity analyses were undertaken to explore the impact of living alone at cohort start fitted as a transition-specific covariate, and frailty status fitted as common-transition effect, adjusted for age, sex, area-level deprivation, and rurality as common effects across transitions and palliative care register as transition-specific. Further sensitivity analyses were undertaken to assess the impact of timing of palliative care registration within 6 months and 1 month before death. Complete data were obtained for all individuals with the exception of eFI. As eFI is unlikely to be missing at random, missing eFI is treated as a category in the analysis to retain all information and account for potentially informative missingness without assuming the data were missing at random. Sensitivity analyses assuming best case (fit) and worst case (severely frail) imputation were used to provide extreme bounds. A data flow diagram is presented in Supplementary Figure S2. All analyses were performed in the SAIL Databank trusted research environment (TRE) using the mstate package^37^^,^^38^ and bespoke code in R version 4.1.3.

### Ethics

Approval for the use of anonymised data in this study, provisioned within the Secure Anonymised Information Linkage (SAIL) Databank, was granted for the purposes of the pre-planned analysis by an independent Information Governance Review Panel (IGRP) under project 1641. The IGRP has a membership comprised of senior representatives from the British Medical Association (BMA), the National Research Ethics Service (NRES), Public Health Wales and Digital Health and Care Wales (DHCW). The usage of additional data was granted by each respective data owner. The SAIL Databank is compliant with General Data Protection Regulations (GDPR) and the UK Data Protection Act. Individual consent for the use of anonymised datasets is not required under UK GDPR.

### Role of the funding source

This study was funded by the Health and Care Research Wales Evidence Centre. The funders of the study had no role in the data collection, analysis, interpretation, writing of the manuscript, or the decision to submit.

## Results

267,199 individuals were included in the analysis. The median age at cohort entry was 81 years (IQR: 72, 88) and 134,046 (50.2%) were female. Overall, 71,151 (26.6%) lived alone at cohort start and 181,243 (67.8%) resided in urban settings. At cohort start, 228,198 (85.4%) were in their own homes, 17,095 (6.4%) were in care homes with nursing, 13,712 (5.1%) were in care homes without nursing, 513 (0.2%) were admitted to hospital with elective admissions, 5365 (2.0%) with emergency admissions, and 2316 (0.9%) with other admissions. Most deaths were due to cancers (76,517, 28.6%) and circulatory diseases (70,725, 26.5%) as the primary cause (Table 1).

**Table 1:** Population characteristics.

	Total	No palliative care register	Palliative care register
	(N = 267,199)	(N = 193,154)	(N = 74,045)
**Age (years)**			
Median [IQR]	81.0 [72, 88]	80.0 [71, 88]	82.0 [72, 89]
**Sex**			
Male	133,153 (49.8%)	98,587 (51.0%)	34,566 (46.7%)
Female	134,046 (50.2%)	94,567 (49.0%)	39,479 (53.3%)
**WIMD quintile**			
1. Most deprived	54,272 (20.3%)	40,712 (21.1%)	13,560 (18.3%)
2	55,110 (20.6%)	40,167 (20.8%)	14,943 (20.2%)
3	54,190 (20.3%)	38,702 (20.0%)	15,488 (20.9%)
4	54,842 (20.5%)	39,038 (20.2%)	15,804 (21.3%)
5. Least deprived	48,785 (18.3%)	34,535 (17.9%)	14,250 (19.2%)
**Rural/urban classification**			
Rural	85,956 (32.2%)	61,111 (31.6%)	24,845 (33.6%)
Urban	181,243 (67.8%)	132,043 (68.4%)	49,200 (66.4%)
**Living alone at cohort start**			
No	196,048 (73.4%)	138,556 (71.7%)	57,492 (77.6%)
Yes	71,151 (26.6%)	54,598 (28.3%)	16,553 (22.4%)
**Electronic frailty index (eFI)**			
Fit	58,265 (21.8%)	41,038 (21.2%)	17,227 (23.3%)
Mild	81,172 (30.4%)	55,649 (28.8%)	25,523 (34.5%)
Moderate	60,269 (22.6%)	40,542 (21.0%)	19,727 (26.6%)
Severe	25,373 (9.5%)	16,094 (8.3%)	9279 (12.5%)
Missing	42,120 (15.8%)	39,831 (20.6%)	2289 (3.1%)
**State at cohort start**			
Care home with nursing	17,095 (6.4%)	10,554 (5.5%)	6541 (8.8%)
Care home without nursing	13,712 (5.1%)	8467 (4.4%)	5245 (7.1%)
Elective admission	513 (0.2%)	325 (0.2%)	188 (0.3%)
Emergency admission	5365 (2.0%)	3517 (1.8%)	1848 (2.5%)
Home	228,198 (85.4%)	168,779 (87.4%)	59,419 (80.2%)
Other admission	2316 (0.9%)	1512 (0.8%)	804 (1.1%)
**Cause of death by ICD-10 chapter**			
Cancers and tumours	76,517 (28.6%)	39,472 (20.4%)	37,045 (50.0%)
Circulatory system diseases	70,725 (26.5%)	61,696 (31.9%)	9029 (12.2%)
Respiratory system diseases	38,847 (14.5%)	31,695 (16.4%)	7152 (9.7%)
Mental health conditions	21,142 (7.9%)	13,359 (6.9%)	7783 (10.5%)
Digestive system diseases	14,472 (5.4%)	13,021 (6.7%)	1451 (2.0%)
Nervous system diseases	13,557 (5.1%)	8539 (4.4%)	5018 (6.8%)
Special cases	8923 (3.3%)	7595 (3.9%)	1328 (1.8%)
Unexplained symptoms	6248 (2.3%)	3975 (2.1%)	2273 (3.1%)
Urinary and reproductive system diseases	5067 (1.9%)	4060 (2.1%)	1007 (1.4%)
Hormone, nutrition, and metabolic disorders	4509 (1.7%)	3520 (1.8%)	989 (1.3%)
Infections and parasites	3025 (1.1%)	2684 (1.4%)	341 (0.5%)
Bone, muscle, and joint disorders	1893 (0.7%)	1599 (0.8%)	294 (0.4%)
Skin conditions	1063 (0.4%)	935 (0.5%)	128 (0.2%)
Birth defects and genetic conditions	590 (0.2%)	483 (0.3%)	107 (0.1%)
Blood and immune system diseases	528 (0.2%)	437 (0.2%)	91 (0.1%)
Other	93 (0.0%)	84 (0.0%)	9 (0.0%)

### Pathways of health and care service utilisation

In total, there were 1,845,572 transitions between all settings in the last year of life. 736,127 (39.9%) transitions were made to a patient's own home, 34,954 (1.8%) transitions to a nursing care home, 29,105 (1.6%) transitions to a care home without nursing, 362,058 (19.6%) transitions to elective admissions, 410,441 (22.2%) transitions to emergency admissions, 5688 (0.3%) to other hospital admissions, and 267,199 (14.5%) transitions to death (Table 2, Supplementary Figure S3). Approximately, half of the transitions to home were from elective admissions (358,522, 48.7%) and half were from emergency admissions (369,597, 50.2%). Of the 34,954 and 29,105 transitions to a care home with and without nursing, 19,534 (55.9%) and 20,605 (70.8%) were from emergency admissions, respectively (Table 2, Supplementary Figure S3). The majority of emergency admissions transitioned from home (370,752, 90.3%), with only 4.5% (18,671) and 5.0% (20,508) transitioning to emergency admissions from care homes with and without nursing, respectively. Similarly, the majority of elective admissions transitioned from home (358,081, 98.9%). Of the 267,199 deaths, 213,126 (79.8%) were from home, 30,866 (11.6%) were from nursing care homes, 17,807 (6.7%) were from care homes without nursing, 5250 (2.0%) were from emergency admissions, 78 (<0.1%) were from elective admissions, and 72 (<0.1%) were from other admissions.

**Table 2:** Frequency (%) of state transitions and restricted expected length of stay (ELOS) and associated 95% confidence intervals (95% CI).

Transition		Frequency	(% of transition to state)	No palliative care register			Palliative care register		
To	From			ELOS	95% CI Lower	Upper	ELOS	95% CI Lower	Upper
Home	Home	736,127	–	320.50	320.50	320.50	304.25	304.25	304.25
Home	Nursing care home	467	0.06	75.91	75.18	76.64	62.51	61.84	63.18
Home	Care home without nursing	445	0.06	112.36	111.48	113.24	93.13	92.31	93.95
Home	Elective admission	358,522	48.70	316.55	316.55	316.55	301.57	301.57	301.57
Home	Emergency admission	369,597	50.21	288.05	288.05	288.05	268.05	268.05	268.05
Home	Other admission	7096	0.96	262.85	262.80	262.90	248.87	248.82	248.92
Nursing care home	Home	11,323	32.39	8.44	8.43	8.45	20.1	20.08	20.12
Nursing care home	Nursing care home	34,954	–	264.33	264.33	264.34	280.93	280.93	280.93
Nursing care home	Care home without nursing	2025	5.79	15.06	14.98	15.14	26.44	26.33	26.55
Nursing care home	Elective admission	1788	5.12	9.94	9.87	10.02	21.11	21.00	21.22
Nursing care home	Emergency admission	19,534	55.88	18.39	18.39	18.40	35.74	35.73	35.75
Nursing care home	Other admission	284	0.81	13.52	12.99	14.06	29.23	28.46	30.00
Care home without nursing	Home	6189	21.26	6.76	6.74	6.77	11.69	11.67	11.71
Care home without nursing	Nursing care home	129	0.44	3.92	3.26	4.58	4.96	4.21	5.71
Care home without nursing	Care home without nursing	29,105	–	208.90	208.90	208.91	222.26	222.25	222.27
Care home without nursing	Elective admission	1867	6.41	7.71	7.65	7.77	12.19	12.11	12.27
Care home without nursing	Emergency admission	20,605	70.80	15.79	15.78	15.80	23.12	23.11	23.13
Care home without nursing	Other admission	315	1.08	13.96	13.51	14.42	21.94	21.37	22.51
Elective admission	Home	358,081	98.90	1.76	1.76	1.76	3.11	3.11	3.11
Elective admission	Nursing care home	1755	0.48	0.62	0.61	0.64	0.86	0.84	0.88
Elective admission	Care home without nursing	1845	0.51	0.87	0.85	0.89	1.24	1.21	1.27
Elective admission	Elective admission	362,058	–	3.34	3.34	3.34	4.36	4.36	4.36
Elective admission	Emergency admission	356	0.10	1.62	1.48	1.77	2.8	2.61	2.99
Elective admission	Other admission	21	0.006	1.53	0	3.96	2.67	0	5.85
Emergency admission	Home	370,752	90.33	26.87	26.87	26.87	25.34	25.34	25.34
Emergency admission	Nursing care home	18,671	4.55	19.80	19.79	19.81	15.48	15.47	15.49
Emergency admission	Care home without nursing	20,508	5.00	27.18	27.17	27.20	21.51	21.50	21.52
Emergency admission	Elective admission	294	0.07	26.79	26.02	27.55	25.26	24.51	26.01
Emergency admission	Emergency admission	410,441	–	40.43	40.43	40.43	34.78	34.78	34.78
Emergency admission	Other admission	216	0.05	25.50	24.46	26.54	23.83	22.83	24.83
Other admission	Home	4854	85.34	0.68	0.67	0.69	0.51	0.50	0.52
Other admission	Nursing care home	161	2.83	0.41	0.23	0.59	0.27	0.13	0.41
Other admission	Care home without nursing	187	3.29	0.62	0.44	0.81	0.41	0.26	0.56
Other admission	Elective admission	22	0.39	0.67	0	2.27	0.5	0	1.87
Other admission	Emergency admission	464	8.16	0.71	0.64	0.79	0.51	0.45	0.57
Other admission	Other admission	5688	–	47.64	47.62	47.65	38.46	38.45	38.47
Death	Home	213,126	79.76	–	–	–	–	–	–
Death	Nursing care home	30,866	11.55	–	–	–	–	–	–
Death	Care home without nursing	17,807	6.66	–	–	–	–	–	–
Death	Elective admission	78	0.03	–	–	–	–	–	–
Death	Emergency admission	5250	1.96	–	–	–	–	–	–
Death	Other admission	72	0.03	–	–	–	–	–	–

### Rurality

Individuals living in urban settings had a 5%, 14%, and 19% increased rate of transition to emergency admissions from home (HR 1.05 [95% CI 1.04–1.06]), nursing care homes (HR 1.14 [95% CI 1.10–1.19]), and care homes without nursing (HR 1.19 [95% CI 1.15–1.24]) respectively compared to those living in rural areas, adjusted for age, sex, area-level deprivation, and palliative care register (Supplementary Table S2). The rate of discharge from emergency settings to home was 4% lower (HR 0.96 [95% CI 0.95–0.97]) for individuals living in urban settings compared to those living in rural settings. The transition rates of admission to nursing care homes were increased for individuals in urban settings compared to those in rural settings from home (HR 1.11 [95% CI 1.06–1.16]), care homes without nursing (HR 1.52 [95% CI 1.38–1.68]), elective admissions (HR 1.61 [95% CI 1.16–2.22]), and other hospital admissions (HR 1.43 [95% CI 1.08–1.90]).

### Palliative care registration

Over the course of the study, 74,045 (27.7%) individuals were on the palliative care register. Of those on the palliative care register, 46,216 (62.4%) were first registered within 6 months before death, and 29,842 (40.3%) were first registered in the last 1 month before death. There appeared to be an under-representation of men (34,566, 46.7%), individuals in the most deprived communities (13,560, 18.3%), living in urban settings (49,200, 66.4%), and living alone (16,533, 22.4%) on the palliative care register compared to those not on the palliative care register. There was an over-representation of individuals in the more severe frailty categories. At cohort entry, 9279 (12.5%) of those on the palliative care register were categorised as severely frail based on the electronic frailty index. The primary cause of death among individuals on the palliative care register was cancer, which made up half of all deaths (37,045, 50.0%) (Table 1).

**Fig. 1:**
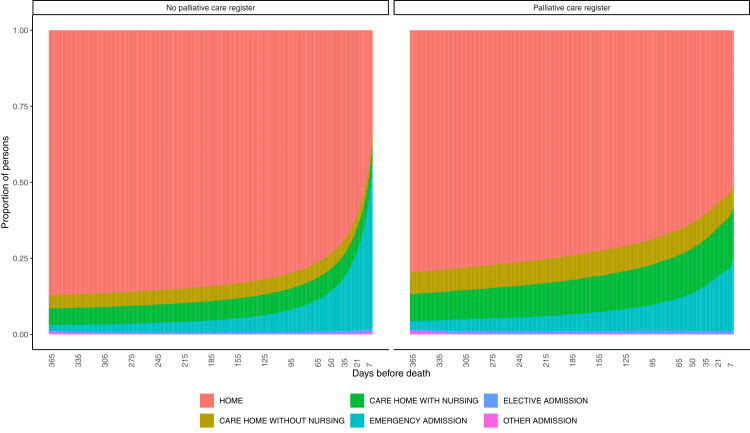
**Proportion of people in each health state in the last year of life by palliative care register status**.

The majority of time in the last year of life was spent at home for both individuals on the palliative care register and those not on the palliative care register (Fig. 1), with an expected length of stay at home of 304.25 (95% CI 304.2497–304.2502) days and 320.50 (95% CI 320.4969–320.4974) days, respectively (Table 2). There were higher proportions of individuals on the palliative care register residing in care homes with and without nursing (Fig. 1), with an expected length of stay of 280.93 (95% CI 280.926–280.934) days and 222.26 (95% CI 222.25–222.27) days for those on the palliative care register, compared to 264.33 (95% CI 264.33–264.34) days and 208.90 (95% CI 208.90–208.91) days for those not on the palliative care register, respectively (Table 2). There was an increased proportion of individuals not in receipt of palliative care using emergency services in the last month of life (Fig. 1, Supplementary Figure S4), with an estimated length of stay of emergency admissions of 40.43 (95% CI 40.430–40.431) days, compared to 34.78 (95% CI 34.779–34.781) days on the palliative care register (Table 2). There appeared to be a consistent trend in the proportion of person-days spent in each health and care service setting between 2015 and 2023 (Supplementary Figure S5).

**Fig. 2:**
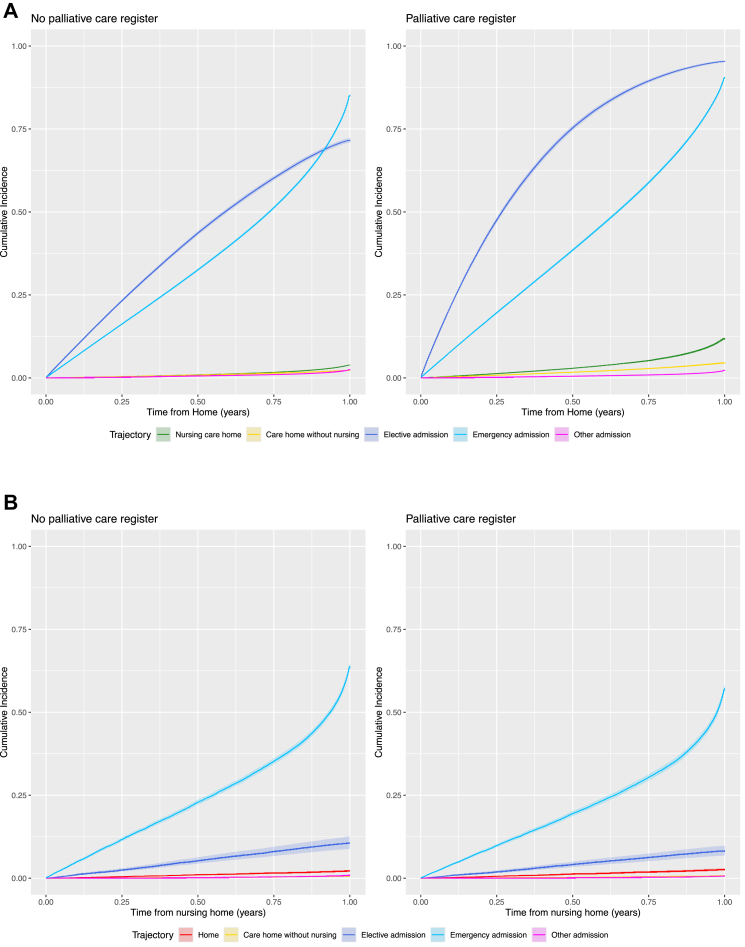

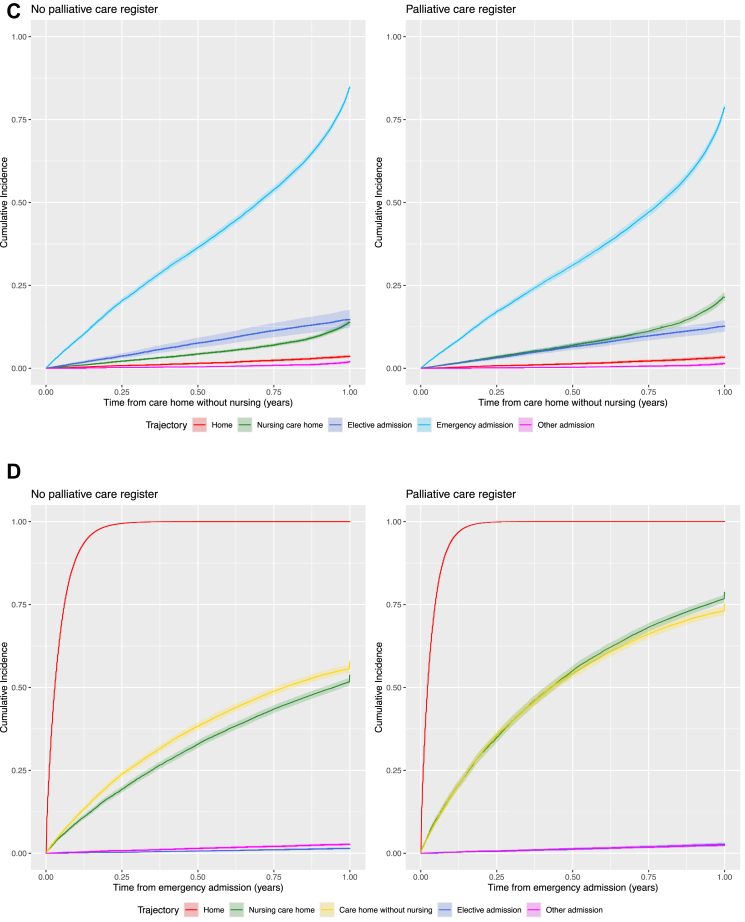

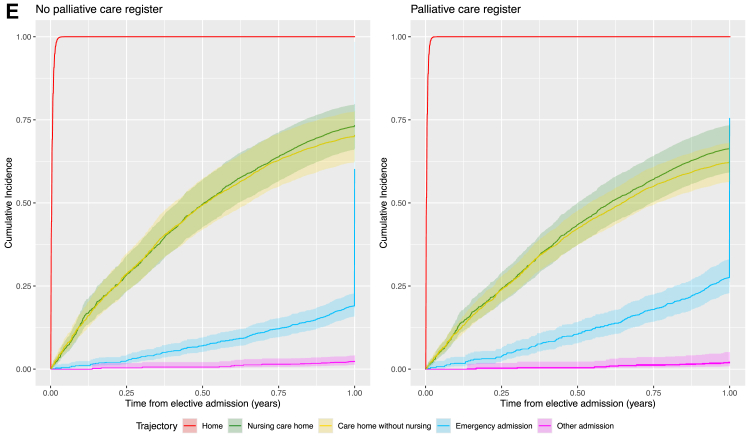
**Cumulative incidence plots based on a 75-year old male living in a most deprived urban community adjusted for rurality and palliative care register. A) Transition from home. B) Transition from nursing care home. C) Transition from care home without nursing. D) Transition from emergency hospital admission. E) Transition from elective hospital admission**.

Fig. 2A shows the cumulative incidence of transitions to health and care service settings in the last year of life from individuals' own homes, stratified by palliative care register status. In both cohorts, emergency and elective hospital admissions were the most frequent events, with cumulative incidence rising steeply in the first few months. Compared with those not on the palliative care register, patients on the register experienced higher cumulative incidence of both emergency and elective admissions, as well as nursing care homes. While moves to care homes without nursing and other hospital admissions were rare in both groups. Those on the palliative care register had an increased rate of transition from home to all other settings in the last year of life, except other admissions where there was a 10% decreased rate in transition (HR 0.90 [95% CI 0.84–0.97]) compared with those not on the palliative care register adjusted for age, sex, area-level deprivation, and rurality (Supplementary Table S2). Individuals on the palliative care register were 3.2 times as likely to transition to a nursing care home (HR 3.20 [95% CI 3.08–3.32]), twice as likely to transition to a care home without nursing (HR 2.02 [95% CI 1.92–2.13]) and 2.4 times as likely to attend an elective admission (HR 2.44 [95% CI 2.37–2.51]) compared with those not on the palliative care register (Fig. 2A). Individuals on the palliative care register had an increased expected length of stay (Table 2) in nursing care homes (ELOS 20.1 days [95% CI 20.08–20.12] vs 8.44 [95% CI 8.43–8.45]), care homes without nursing (ELOS 11.69 days [95% CI 11.67–11.71] vs 6.76 [95% CI 6.74–6.77]) and elective admissions (ELOS 3.11 days [95% CI 3.1098–3.1102] vs 1.76 [95% CI 1.7597–1.760]) from home compared with those not on the palliative care register. Transitions from the patient's own home setting to emergency admissions were 23% higher for those on the palliative care register compared with those not on the register (HR 1.23 [95% CI 1.22–1.25]). However, those on the palliative care register had a decreased expected length of stay in emergency admissions from home (ELOS 25.34 days [95% CI 25.339–25.3406] vs 26.87 [95% CI 26.867–26.878]). The rate of emergency admissions from nursing care homes (Fig. 2B) and care homes without nursing (Fig. 2C) were 17% (HR 0.83 [95% CI 0.80–0.86]) and 18% (HR 0.82 [95% CI 0.79–0.85]) lower for individuals on the palliative care register compared with those not on the register (Supplementary Table S2), with a decreased expected length of stay in emergency admissions from nursing care homes (ELOS 15.48 [95% CI 15.47–15.49] vs 19.80 [95% CI 19.79–19.81]) and care homes without nursing (ELOS 21.51 [95% CI 21.50–21.52] vs 27.18 [95% CI 27.17–27.20]), respectively (Table 2). For individuals admitted to emergency care on the palliative care register, there were increased rates of transition to home (HR 1.27 [95% CI 1.25–1.28]), nursing care home (HR 2.00 [95% CI 1.92–2.09]), care home without nursing (HR 1.62 [95% CI 1.54–1.69]), and elective admission (HR 1.83 [95% CI 1.42–2.35]) compared with those not on the palliative care register (Fig. 2D, Supplementary Table S2). However, individuals on the palliative care register had an increased expected length of stay in nursing care homes (ELOS 35.74 [95% CI 35.73–35.75] vs 18.39 [95% CI 18.39–18.40]), care homes without nursing (ELOS 23.12 [95% CI 23.11–23.13] vs 15.79 [95% CI 15.78–15.80]) and elective admissions (ELOS 2.8 [95% CI 2.61–2.99]) from emergency admissions, but a decreased expected length of stay at home (ELOS 268.05 [95% CI 268.049–268.051] vs 288.047 [95% CI 288.047–288.048]) from emergency admissions compared with those not on the palliative care register (Table 2). Similarly, elective admissions were discharged to home at a 24% (HR 1.24 [95% CI 1.20–1.28]) increased rate (Fig. 2E, Supplementary Table S2) but with a decreased expected length of stay at home (ELOS 301.57 [95% CI 301.569–301.571] vs 316.553 [95% CI 316.552–316.553]) for palliative care registered patients compared with those not on the palliative care register. Results were robust for different ages (Supplementary Figure S6), sex (Supplementary Figure S7), area-level deprivation (Supplementary Figure S8), rurality (Supplementary Figure S9), frailty status (Supplementary Figures S10 and S11), and those living alone at cohort start (Supplementary Figure S12). Results were also robust to timing of palliative care registration with similar trends found for those first registered for palliative care within 6 months (Supplementary Figure S13) and 1 month (Supplementary Figure S14) before death.

## Discussion

This population-scale time-resolved analysis has identified that the majority of the last year of life for those who die from non-sudden causes was spent at home, however, there is an increased demand on urgent care settings towards the end-of-life, with 90.3% of all emergency admissions transitioning from home. This finding is congruent with existing literature summarising end-of-life transitions in Belgium, the Netherlands, Italy and Spain, which found an increased proportion of transitions to hospital settings for people residing at home.^15^ Overall, 26.6% of individuals lived alone in the last year of life, which is likely to represent additional complexity of care at the end-of-life.

A large proportion of admissions to care homes with and without nursing were from emergency admissions, necessitating the need for efficient pathways of care to community-based care settings, where it is appropriate to do so, to alleviate the pressures faced in emergency departments and on admissions in general, to minimise unnecessary disruption for patients.

Residents from urban areas had an increased rate of emergency hospital admissions and nursing home admissions from non-hospital settings, which may be related to the availability and closer proximity of health and care resources in urban settings and/or potentially stronger wrap around community support in rural communities. This finding is in keeping with a study in Northern Ireland, which found an increased rate of emergency admissions in the last year of life for urban residents dying of cancer.^22^ In our study, urban residents also had a lower rate of discharge from emergency hospital admissions to home compared to those living in rural settings. This may be related to the organisation of community services within rural areas aimed at supporting individuals within their own home and/or support from family and care-providers. However, a recent study found that there was substantial variation in community nursing support by geographical region in a similar UK population, with a lower workforce provision per 100,000 population in rural areas and a higher workforce provision in more deprived communities.^39^ Rural areas in Wales are mostly classified in the 4th and 5th quintiles of the Welsh Index of Multiple Deprivation, representing some of the least deprived communities in Wales.^28^ It is unclear whether district nursing provision follows a similar pattern to that reported in similar UK populations since health and social care services are devolved. Furthermore, a recent study in Belgium, the Netherlands and England, found that there were more hospital beds and care home beds in urban populations compared to rural populations, with the exception of England where there were more care home beds available in rural areas compared to urban areas.^40^ Household compositions may also play an important role in transitions to and from health and care settings, where single occupancy households may result in increased health and care service utilisation. However, in our study, sensitivity analyses evaluating the impact of living alone found similar trends. A previous study reported that urban populations in England had fewer one-person households for individuals over 65 years compared to more rural populations, whilst the opposite was true for the Netherlands and Belgium.^40^ Future work could explore whether health and social care workforce, household composition, and proximity to health and care services, together with their intersectionality, affect the rate of admission and discharge by rural-urban classification.

Individuals in receipt of palliative care were more likely to transition to a nursing care home, care home without nursing, elective admission, and emergency admission from home, compared with those not on the palliative care register, adjusted for age, sex, area-level deprivation, and rurality. However, the rate of emergency admissions from nursing care homes and care homes without nursing were lower for individuals on the palliative care register compared to those not on the palliative care register. Similarly, the rates of discharge from emergency, elective and other admissions to non-hospital settings were increased for those on the palliative care register, resulting in a decreased expected length of stay for emergency and other admissions. These findings suggest that there was more effective management of palliative care patients from home and urgent settings to community care/nursing facilities, demonstrating the importance of efficient identification of individuals in need of palliative care services to ensure appropriate management and care for those nearing the end-of-life. These findings are in keeping with a recent study in the USA evaluating hospital length of stay, which reported a reduced length of stay for individuals receiving speciality palliative care consultations compared to propensity matched controls.^7^ However, in patients with chronic obstructive pulmonary disease, it has previously been reported that there was no difference in days spent at home, and healthcare use for those with and without palliative care, but there was an increased rate of hospitalisations for those receiving palliative care.^13^ The latter finding is in keeping with the findings reported in this study for a broader population nearing the end-of-life, where palliative care patients residing in homes were more likely to experience hospital admissions.

Only 27.7% of individuals were registered as in receipt of palliative care in the last year of life. There appeared to be an under-representation of men, residents from urban areas, those living in the most-deprived communities and those living alone on the palliative care register, which may exacerbate potential health inequalities. These findings are in keeping with a recent study in the USA, which found that unpartnered individuals were less likely to attend an outpatient palliative care visit, and men were less likely to engage in further palliative care follow-up.^12^ Future work could assess the impact of these potential health inequalities on patient outcomes and explore opportunities to promote the identification of eligible patients in under-represented groups. One such approach is the development of prediction models to more accurately identify those needing palliative care services who are nearing the end-of-life.^18^

Previous studies in the UK^23^^,^^24^ have found that hospital costs were the largest costs associated with end-of-life care. A public expenditure report in the UK has found that of the public funds spent on healthcare for individuals in the last year of life, an estimated 81% were spent in hospital settings, and 56% were spent in emergency hospital care.^24^ A previous body of work has also shown that home-based interventions may offer cost savings for the health system and improved outcomes for patients.^41^ As the majority of time in the last year of life was spent at home, and there was an increased rate of admissions to emergency care from home, providing additional support at home and facilitating appropriate management outside of urgent care settings, where it is appropriate to the patient's needs and preferences, has the potential to optimise health service provision and reduce costs.

This study has identified transition patterns that may reflect potentially low-value care, such as multiple urgent hospitalisations in the final days of life or transitions close to death. These transitions have previously been described as burdensome transitions,^42^ and may reflect missed opportunities for timely palliative care or end-of-life care planning. Future work should explore these transitions in more detail, particularly in the context of health system interventions aimed at reducing low-value care, enhancing patient-reported outcome measures such as quality of life/quality of death, and meeting patients' preferences. Previous work in four European countries has identified that patient and/or family preference was frequently cited as the reason for many hospitalisations.^15^ The palliative care register appeared to be associated with provision of higher-value care for patients and the health service compared with those not on the palliative care register, by reducing urgent hospitalisations in the final days of life and/or transitions close to death. However, these transitions may not necessarily reflect poor quality of care. Such transitions could occur for clinically appropriate reasons, including symptom control, acute illness unrelated to the terminal condition, or injury management (e.g., fracture). Our analysis examines patterns of service utilisation rather than directly measuring quality. Future research should validate whether transitions represent potentially burdensome care, for example by linking to bereaved family member reports or other established quality indicators. These findings support the case for potential investment in improving quality and capacity of palliative services including evaluation of appropriate care management.

Care homes may be more likely to consider whether residents are eligible for palliative care services and initiate the process of registration (for palliative care) with General Practitioners compared to those at home, who may be less systematically identified. Similarly, individuals are more likely to be considered for palliative care services following an urgent hospital admission and/or discharge notification. Care home admissions are also more likely among individuals on the palliative care register, which may reflect increased identification of care needs. Whilst we adjusted for several factors that influence access to palliative care and health and care service utilisation including age, sex, area-level deprivation, and rurality, we acknowledge that unmeasured confounders such as clinician referral patterns, patient preferences, or local service availability may still influence palliative care registration and health and care service utilisation. As such, differences in healthcare utilisation by palliative care status may partly reflect these unmeasured access disparities. The multi-state modelling approach only allows us to make inferences regarding the association between palliative care registration and health and care service utilisation. Further work should explore potential causal pathways using causal inference methods such as inverse probability weighting, pseudo-observations or G-formula.^43^

A potential limitation of using routinely collected EHR data is the appropriate coding of individuals in receipt of palliative care services. In this analysis, we used Read codes that are used by the Quality Outcomes Framework with additional codes recommended by the Gold Standard Framework.^32^ A target of the Quality Outcomes Framework was to hold a register of palliative care patients, and to review patients with the local palliative care team on a regular basis. This may typically involve monthly meetings to review patients on the palliative care register and discuss their potential needs. Although general practices across Wales have different processes for managing palliative care registered individuals, the findings of this study indicate that initiating any such process of holding a palliative care register with regular review is associated with higher-value care for patients and the health service. Completeness of reporting of palliative care registers also varies by General Practice, and as a result, we may not have captured everyone in receipt of palliative care services, leading to an under-representation of those on the palliative care register. However, under registration of those in receipt of palliative care is likely to be associated with under provision of palliative care services and as a result are unlikely to meaningfully impact the findings of this study. For computational efficiency (and difficulties in identifying cessation of palliative care in routine data), palliative care status is not assumed to vary with time (i.e., it is not fitted as a time-varying covariate in the model), which may lead to an over-representation of those on the palliative care register at any point in time, and as such may mis-classify individuals resulting in an attenuation of the difference in transition rates from those on the palliative care register compared with those not in receipt of palliative care services. However, findings from sensitivity analyses evaluating those on the palliative care register in the last 6-months and 1-month before death were robust. We also explored the robustness of results to frailty status in sensitivity analyses using the eFI. The eFI can be considered a poor measure of frailty and has previously been found to overestimate frailty status^44^ and thus sensitivity analyses with respect to frailty status should be interpreted with caution. The eFI2 is likely to be a better indicator of frailty status on average compared to the eFI, however external validation of the eFI2 suggests that high risk individuals may be underpredicted and low risk individuals may be overpredicted.^45^

A limitation of the existing data is the absence of information related to hospice care services, which is not routinely available through the SAIL Databank NHS data collections. In this study, home addresses as recorded in the WDSD were used as place of residence, meaning periods of time spent in a hospice which were not captured either as a change of address or linked to a residential care home identifier were treated as time at home. Understanding the transitions between all settings, including hospices would provide further valuable insights. As linked data become increasingly available, the modelling framework could easily be extended to differentiate between other settings including home with and without home care packages and hospice services provided by non-NHS organisations. Electronic health and administrative data sources also do not routinely collect information on important outcomes including quality of life and patient preferences, prohibiting the analysis of patient reported outcomes such as differences in quality of life, quality of death, and/or expectations/satisfaction of care. Future work could also evaluate the impact of clinical diagnosis on health and care pathways. In this paper, we summarise the causes of death, however, it is statistically inappropriate to adjust for cause of death in the modelling framework as we would be conditioning on a future event,^46^ leading to potential temporal misclassification which may distort effect estimates of interest. Future work could model cause-specific death states in a multi-state model, however, in our example, this would add a substantial number of states to the multi-state model resulting in computational difficulties.

The statistical analysis was adjusted for age, sex, area-level deprivation, rurality and palliative care register. Age, sex, and area-level deprivation were assumed to have common effects across transitions for computational stability, whilst rurality and palliative care register were assumed to have transition-specific effects. The assumption of a common effect for age, sex, and area-level deprivation for each transition may be an over-simplification. Future work should apply these modelling frameworks to larger populations, with increased numbers of transitions for different covariate profiles to relax this assumption. It was not possible to replicate this study in other jurisdictions in the UK owing to a lack of availability of population-scale linked EHR and administrative data.^47^ As these data become available, future work could externally validate these findings in other UK jurisdictions.

This work enabling a population-scale system-wide evaluation was only made possible by linkage of EHR and administrative data across the entire population of Wales facilitated by the SAIL Databank.^25^ As the availability of social care data increases,^48^ future work could link health and administrative data to social care records to further understand resource use and support provided from social care services. This could include, for example, understanding the use and provision of home care packages in the last year of life and quantifying how social care services support and facilitate time spent at home.

The findings from this study could be used as a benchmark with which to evaluate the potential impact of end-of-life and palliative care policy recommendations on health and care services in a UK population. Future work could simulate alternative scenarios of potential interventions and/or service redesign to help inform health policy decision-making and practice.

In summary, this population-scale analysis found that there was differential uptake and length of stay in health and care services for those in rural areas and those on the palliative care register. Whilst the majority of time the last year of life was spent at home, there was increased demand on urgent care settings towards the end-of-life. To optimise healthcare resources, health and care systems should prioritise identifying individuals requiring palliative care and providing additional support at home. The findings from this study can be used as a benchmark with which to evaluate evolving policy recommendations for those nearing the end-of-life.

## Contributors

Conceptualisation of the study: RKO, RB, IB; Data curation and access to raw data: RKO, RB, AM, HD, AA; Data verification: RKO, RB; Analysis: RKO, RB; Drafting of the paper: RKO; Review, editing and final decision to submit the manuscript: RKO, RB, HD, AM, AA, EC, AC, NJW, AE, MP, IB.

## Data sharing statement

Access to the data used for all analyses, figures and tables are available to the research community upon approval by the SAIL Databank (https://saildatabank.com/data/apply-to-work-with-the-data/). All proposals to use data held within the SAIL Databank are subject to review and approval by an independent Information Governance Review Panel (IGRP).

## Declaration of interests

RKO is a member of the National Institute for Health and Care Excellence (NICE) Technology Appraisal Committee, member of the NICE Decision Support Unit (DSU), and associate member of the NICE Technical Support Unit (TSU). She has served as a paid consultant to the pharmaceutical industry, providing unrelated methodological advice. She reports teaching fees from the Association of British Pharmaceutical Industry (ABPI) and the University of Bristol. All other authors have no declarations of interest.
