# Supplementary material for: Health and care service utilisation in the last year of life before non-sudden death in Wales, 2014–2023, by palliative care registration: a population-based retrospective cohort study
Source: Lancet Reg Health Eur. 2025 Oct 7;59:101479. doi: 10.1016/j.lanepe.2025.101479 (PMC12538943; doi:10.1016/j.lanepe.2025.101479)

Supplementary Material

Table of Contents

Supplementary Table 1: Palliative Care Register Read Codes v2 3

Supplementary Figure 1: Multi-state Model... 3

Supplementary Figure 2: Data flow diagram 4

Supplementary Figure 3: Sankey Flow diagram illustrating the trajectories of health and care service pathways 5

Supplementary Table 2: Coefficients obtained from Multi-state Cox Proportional Hazards Model 6

Supplementary Figure 4: Proportion of individuals in each health and care service setting in the last 1 month of life. 11

Supplementary Figure 5: Trends in health and care service utilisation, and death, between 2015 and 2023...12

Supplementary Figure 6: Cumulative incidence plots for ages 25 and 50 adjusted for sex, area-level deprivation, rurality, and palliative care register 13

Supplementary Figure 7: Cumulative incidence plots for females adjusted for age, area-level deprivation, rurality, and palliative care register.................................16

Supplementary Figure 8: Cumulative incidence plots for least deprived adjusted for age, sex, rurality, and palliative care register..19

Supplementary Figure 9: Cumulative incidence plots for rural areas adjusted for age, sex, area-level deprivation, and palliative care register 22

Supplementary Figure 10: Sensitivity analysis - cumulative incidence plots for frailty adjusted for age, sex, rurality, frailty status, and area-level deprivation as fixed covariates, and palliative care register as transition specific covariates.......25

Supplementary Figure 11: Sensitivity analysis - cumulative incidence plots for frailty assuming worst case (all missing data are severely frail) and best case (all missing data are fit) adjusted for age, sex, rurality, and area-level deprivation as fixed covariates, and palliative care register as transition specific covariates.......28

Supplementary Figure 12: Sensitivity analysis - cumulative incidence plots for living alone, adjusted for age, sex, rurality, and area-level deprivation as fixed covariates, and palliative care register and living alone as transition specific covariates 31

Supplementary Figure 13: Sensitivity analysis - cumulative incidence plots for palliative care registration within 6 months of death, adjusted for age, sex, and area-level deprivation as fixed covariates, and rurality and palliative care register as transition specific covariates 34

Supplementary Figure 14: Sensitivity analysis - cumulative incidence plots for palliative care registration within 1 month of death, adjusted for age, sex, and area-level deprivation as fixed covariates, and rurality and palliative care register as transition specific covariates 37

Supplementary Table 1: Palliative Care Register Read Codes v2

8BAP.	8BAe.	8BAS.	8BAT.	8BA2.	8H6A.
8CM1%*	8CM4.	8CMQ.	8CMb.	8HH7.	8IEE.
8BJ1.	8H7L.	8H7g.	9EB5.	1Z01.	9NNf0
ZV57C	2JE..	8CME.	9NgD.	9Ng7.	9G8..
9c0P.	9c0N.	8CMW3	9K9..	9367.	9c0L0
9c0M.	9NND.	8HY..	8BAR.	8BAQ.	

*Excluding 8CM15 as life expectancy is >1 year.

Supplementary Figure 1: Multi-state Model

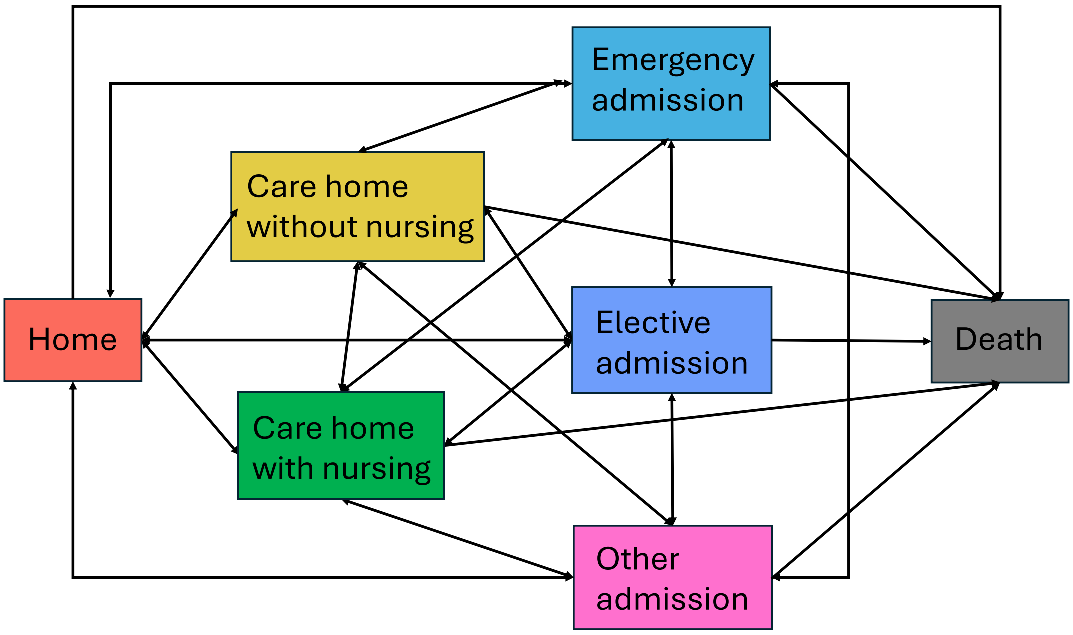

Supplementary Figure 2: Data flow diagram

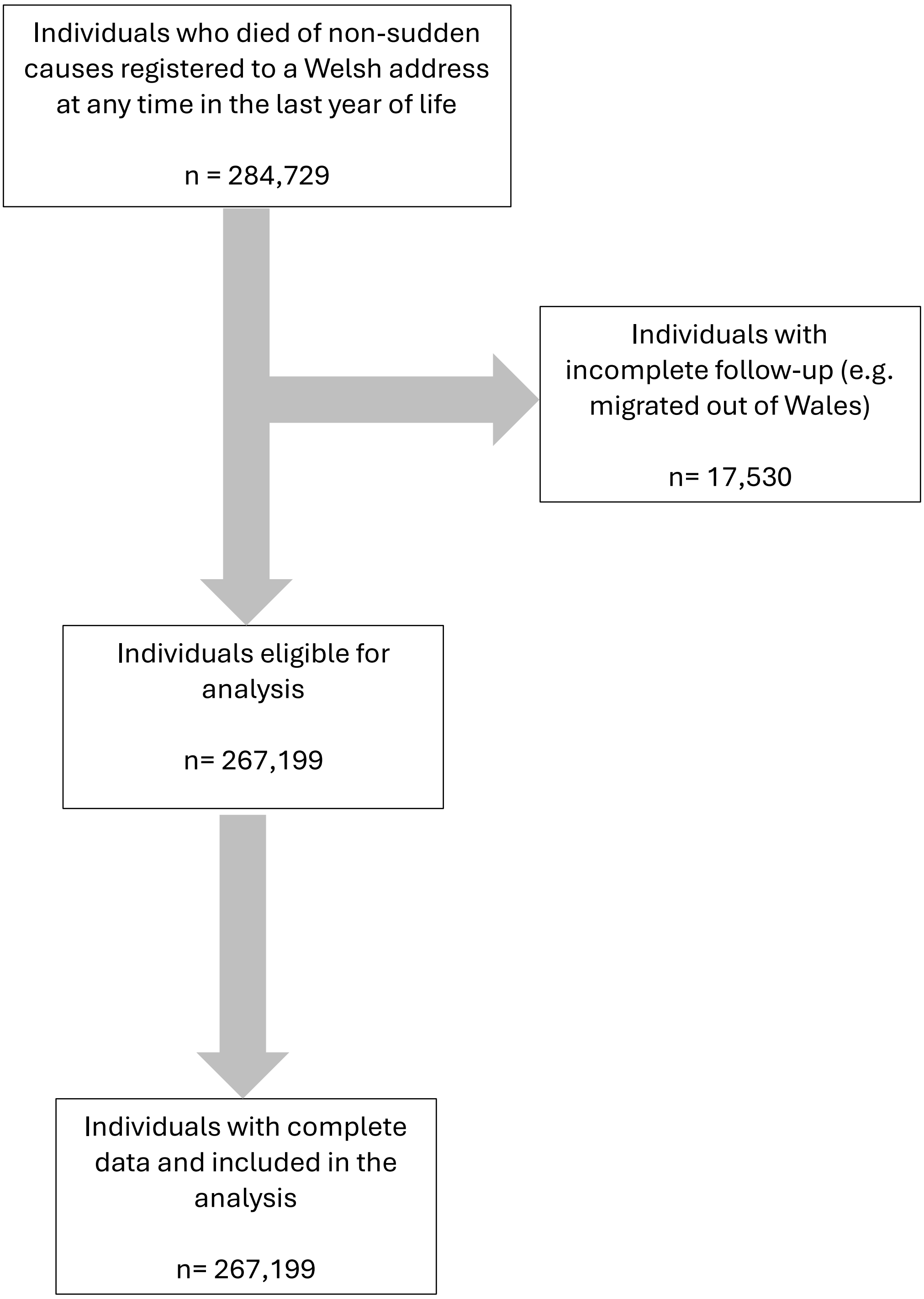

Supplementary Figure 3: Sankey Flow diagram illustrating the trajectories of health and care service pathways

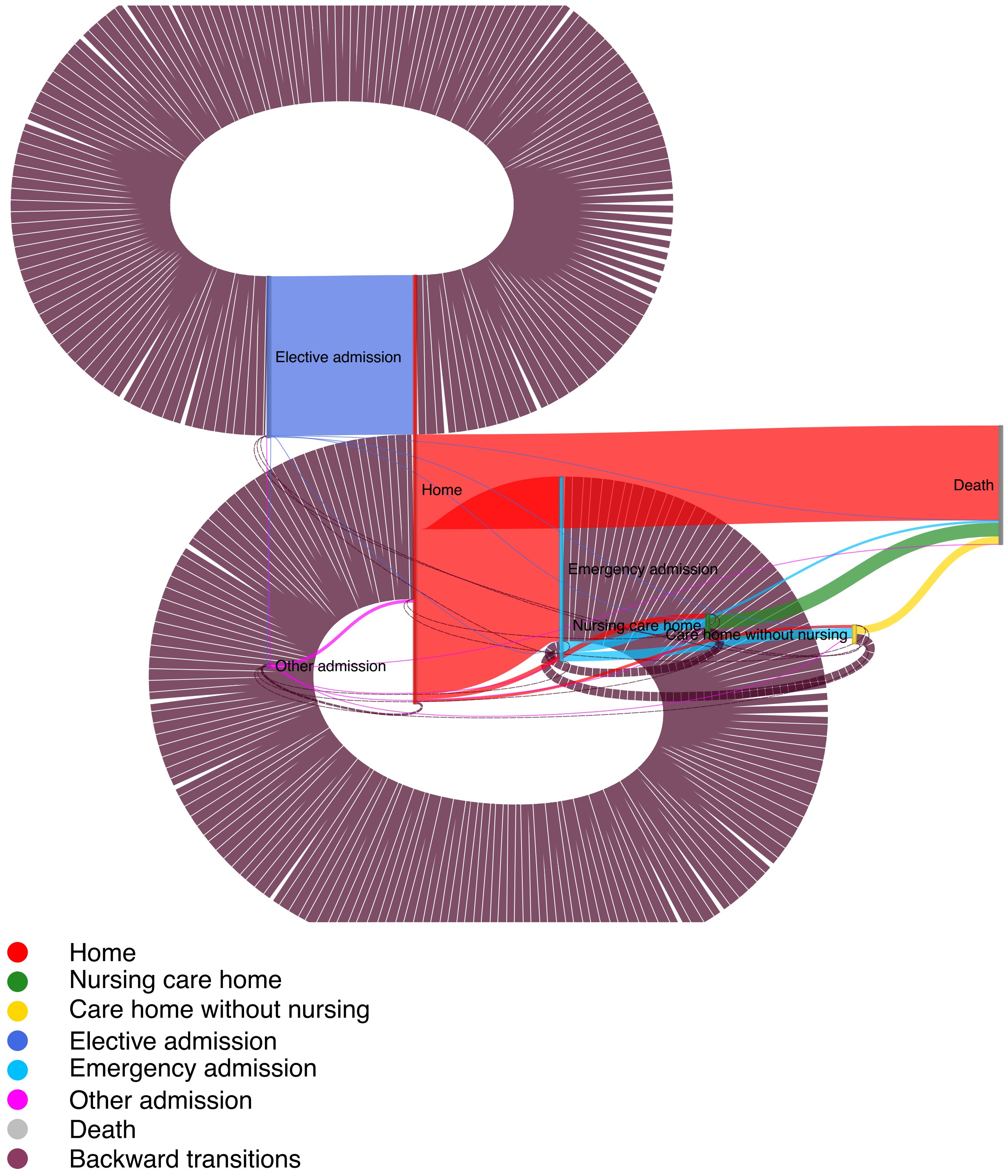

Supplementary Table 2: Coefficients obtained from Multi-state Cox Proportional Hazards Model

Coefficient	Transition	Hazard Ratio	95% Confidence Interval	
Age	-	0.99	0.99	0.99
Sex: Female	-	0.98	0.97	0.99
WIMD 2nd quintile	-	1.01	1.00	1.03
WIMD 3rd quintile	-	1.02	1.01	1.04
WIMD 4th quintile	-	1.06	1.04	1.07
WIMD 5th quintile: Least Deprived	-	1.07	1.05	1.09
Urban	Home > Nursing care home	1.11	1.06	1.16
Urban	Home > Care home without nursing	0.86	0.81	0.90
Urban	Home > Elective admission	1.02	0.99	1.06
Urban	Home > Emergency admission	1.05	1.04	1.06
Urban	Home > Other admission	0.86	0.81	0.92
Urban	Home > Death	0.99	0.98	0.99
Urban	Nursing care home > Home	0.87	0.71	1.07
Urban	Nursing care home > Care home without nursing	0.67	0.46	0.96
Urban	Nursing care home > Elective admission	1.23	0.89	1.69
Urban	Nursing care home > Emergency admission	1.14	1.10	1.19
Urban	Nursing care home > Other admission	1.07	0.74	1.54
Urban	Nursing care home > Death	1.01	1.00	1.01
Urban	Care home without nursing > Home	1.02	0.84	1.25

Urban	Care home without nursing > Nursing care home	1.52	1.38	1.68
Urban	Care home without nursing > Elective admission	1.07	0.79	1.44
Urban	Care home without nursing > Emergency admission	1.19	1.15	1.24
Urban	Care home without nursing > Other admission	1.81	1.28	2.56
Urban	Care home without nursing > Death	1.00	0.99	1.00
Urban	Elective admission > Home	0.99	0.96	1.02
Urban	Elective admission > Nursing care home	1.61	1.16	2.22
Urban	Elective admission > Care home without nursing	0.90	0.66	1.22
Urban	Elective admission > Emergency admission	1.35	0.99	1.84
Urban	Elective admission > Other admission	2.27	0.75	6.90
Urban	Elective admission > Death	0.94	0.88	1.02
Urban	Emergency admission > Home	0.96	0.95	0.97
Urban	Emergency admission > Nursing care home	1.34	1.28	1.41
Urban	Emergency admission > Care home without nursing	0.91	0.88	0.96
Urban	Emergency admission > Elective admission	0.86	0.67	1.10
Urban	Emergency admission > Other admission	0.82	0.67	1.00
Urban	Emergency admission > Death	0.98	0.97	1.00
Urban	Other admission > Home	0.85	0.79	0.91

Urban	Other admission > Nursing care home	1.43	1.08	1.90
Urban	Other admission > Care home without nursing	1.37	1.04	1.79
Urban	Other admission > Elective admission	0.84	0.33	2.14
Urban	Other admission > Emergency admission	0.89	0.66	1.21
Urban	Other admission > Death	0.98	0.88	1.09
Palliative care register	Home > Nursing care home	3.20	3.08	3.32
Palliative care register	Home > Care home without nursing	2.02	1.92	2.13
Palliative care register	Home > Elective admission	2.44	2.37	2.51
Palliative care register	Home > Emergency admission	1.23	1.22	1.25
Palliative care register	Home > Other admission	0.90	0.84	0.97
Palliative care register	Home > Death	1.00	1.00	1.00
Palliative care register	Nursing care home > Home	1.21	1.00	1.46
Palliative care register	Nursing care home > Care home without nursing	1.13	0.79	1.60
Palliative care register	Nursing care home > Elective admission	0.77	0.55	1.09
Palliative care register	Nursing care home > Emergency admission	0.83	0.80	0.86
Palliative care register	Nursing care home > Other admission	0.71	0.51	1.00
Palliative care register	Nursing care home > Death	1.00	1.00	1.00
Palliative care register	Care home without nursing > Home	0.92	0.76	1.12
Palliative care register	Care home without nursing > Nursing care home	1.64	1.50	1.79
Palliative care register	Care home without nursing > Elective admission	0.85	0.63	1.15

Palliative care register	Care home without nursing > Emergency admission	0.82	0.79	0.85
Palliative care register	Care home without nursing > Other admission	0.74	0.53	1.02
Palliative care register	Care home without nursing > Death	1.02	1.02	1.03
Palliative care register	Elective admission > Home	1.24	1.20	1.28
Palliative care register	Elective admission > Nursing care home	0.83	0.58	1.19
Palliative care register	Elective admission > Care home without nursing	0.81	0.58	1.12
Palliative care register	Elective admission > Emergency admission	1.53	1.16	2.02
Palliative care register	Elective admission > Other admission	0.91	0.33	2.49
Palliative care register	Elective admission > Death	1.00	0.91	1.10
Palliative care register	Emergency admission > Home	1.27	1.25	1.28
Palliative care register	Emergency admission > Nursing care home	2.00	1.92	2.09
Palliative care register	Emergency admission > Care home without nursing	1.62	1.54	1.69
Palliative care register	Emergency admission > Elective admission	1.83	1.42	2.35
Palliative care register	Emergency admission > Other admission	0.91	0.73	1.12
Palliative care register	Emergency admission > Death	1.01	0.99	1.02
Palliative care register	Other admission > Home	1.18	1.10	1.28
Palliative care register	Other admission > Nursing care home	2.25	1.75	2.89
Palliative care register	Other admission > Care home without nursing	1.70	1.32	2.18

Palliative care register	Other admission > Elective admission	0.72	0.25	2.10
Palliative care register	Other admission > Emergency admission	1.08	0.78	1.50
Palliative care register	Other admission > Death	1.08	0.91	1.27

Supplementary Figure 4: Proportion of individuals in each health and care service setting in the last 1 month of life.

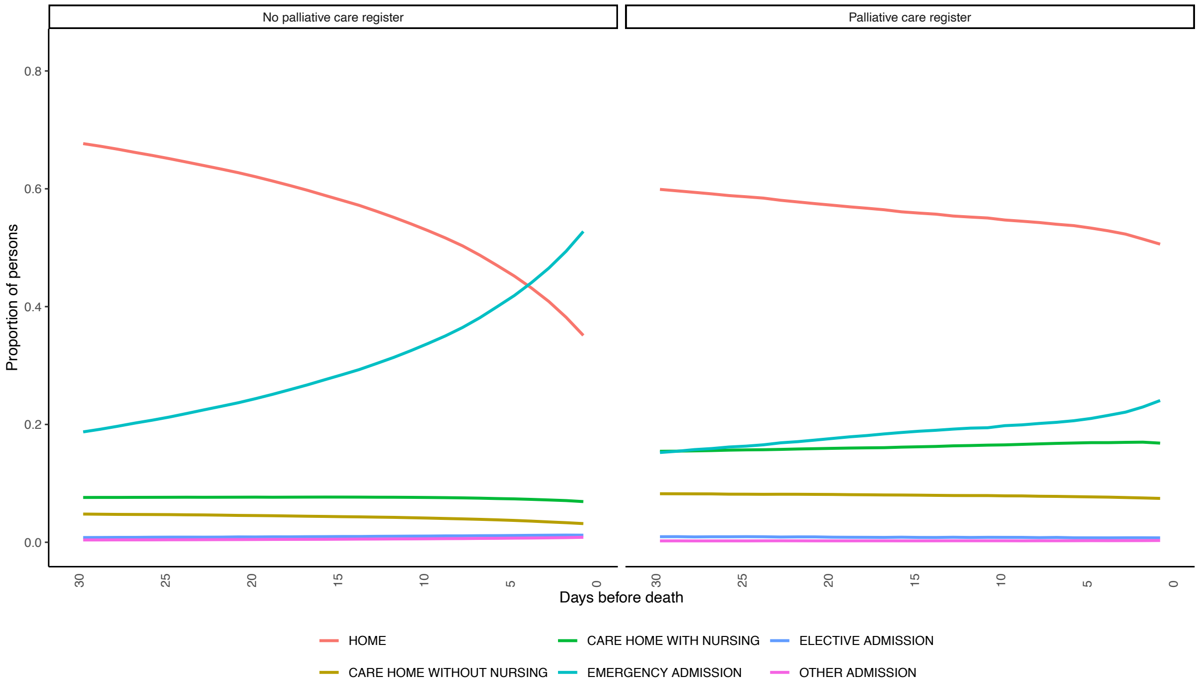

Supplementary Figure 5: Trends in health and care service utilisation, and death, between 2015 and 2023.

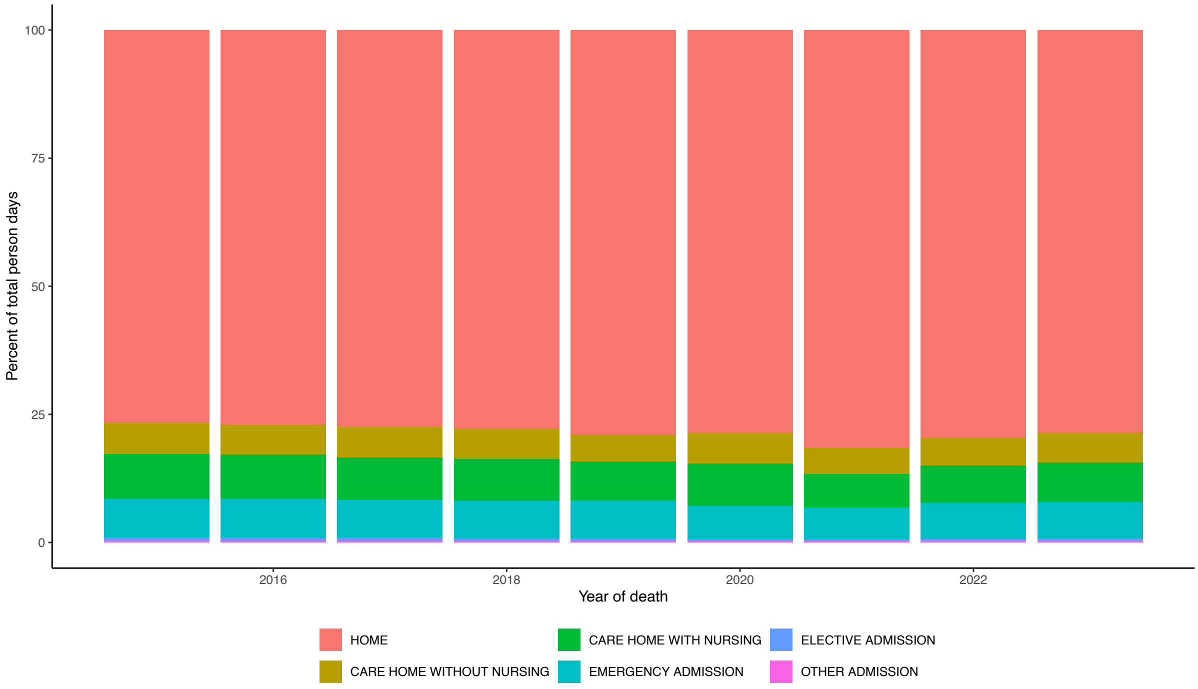

Supplementary Figure 6: Cumulative incidence plots for ages 25 and 50 adjusted for sex, area-level deprivation, rurality, and palliative care register

i) Transitions from home

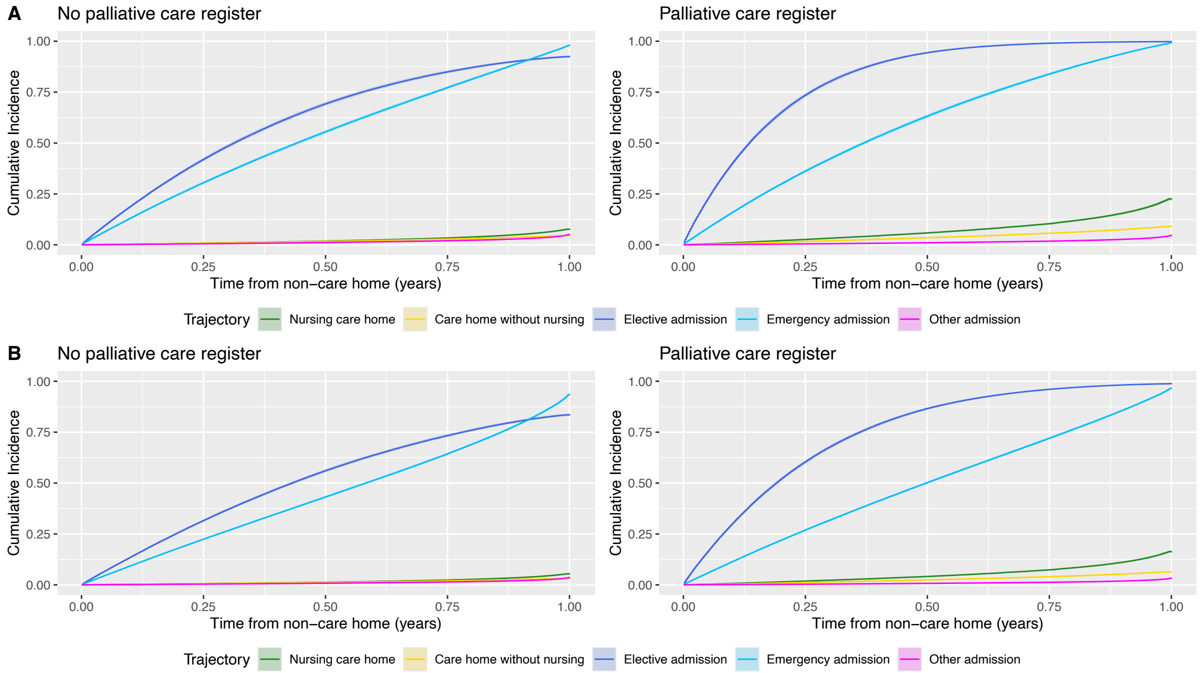

A: 25 years; B: 50 years

ii) Transitions from nursing care home

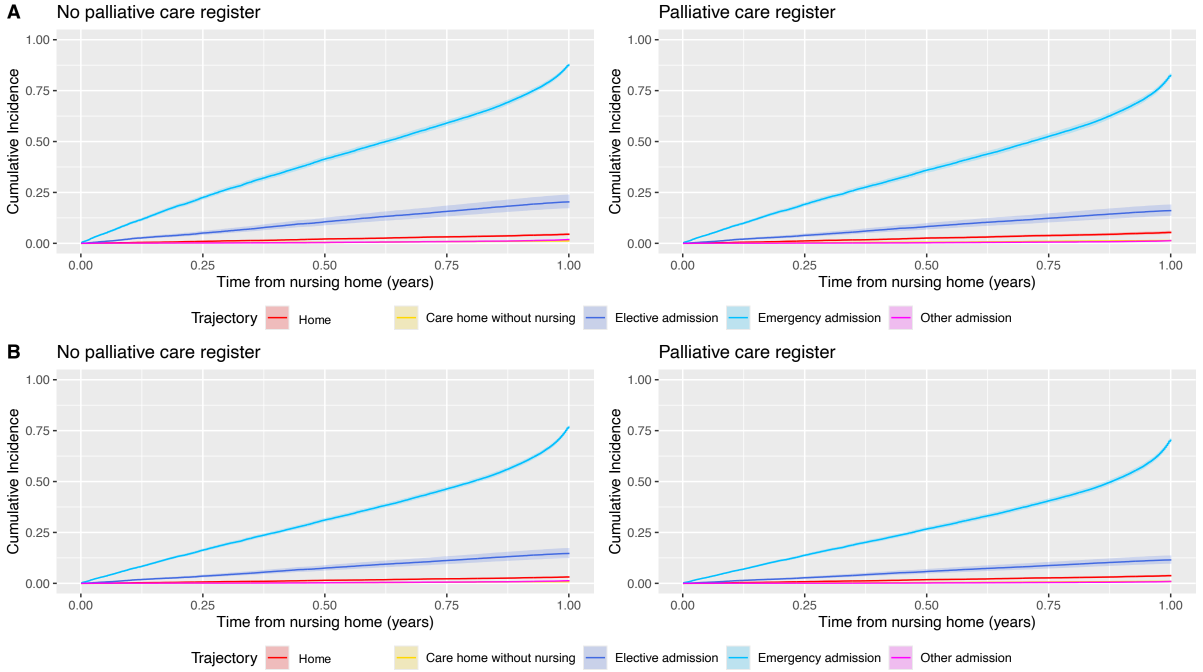

A: 25 years; B: 50 years

iii) Transitions from care home without nursing

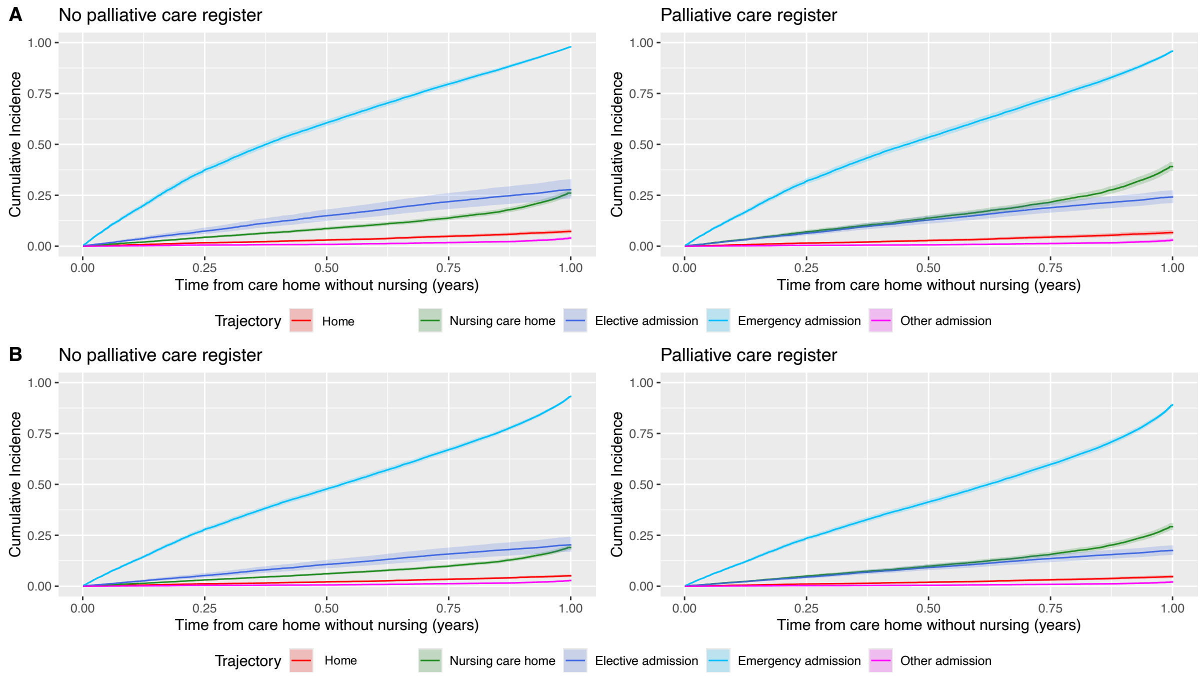

A: 25 years; B: 50 years

iv) Transitions from emergency hospital admission

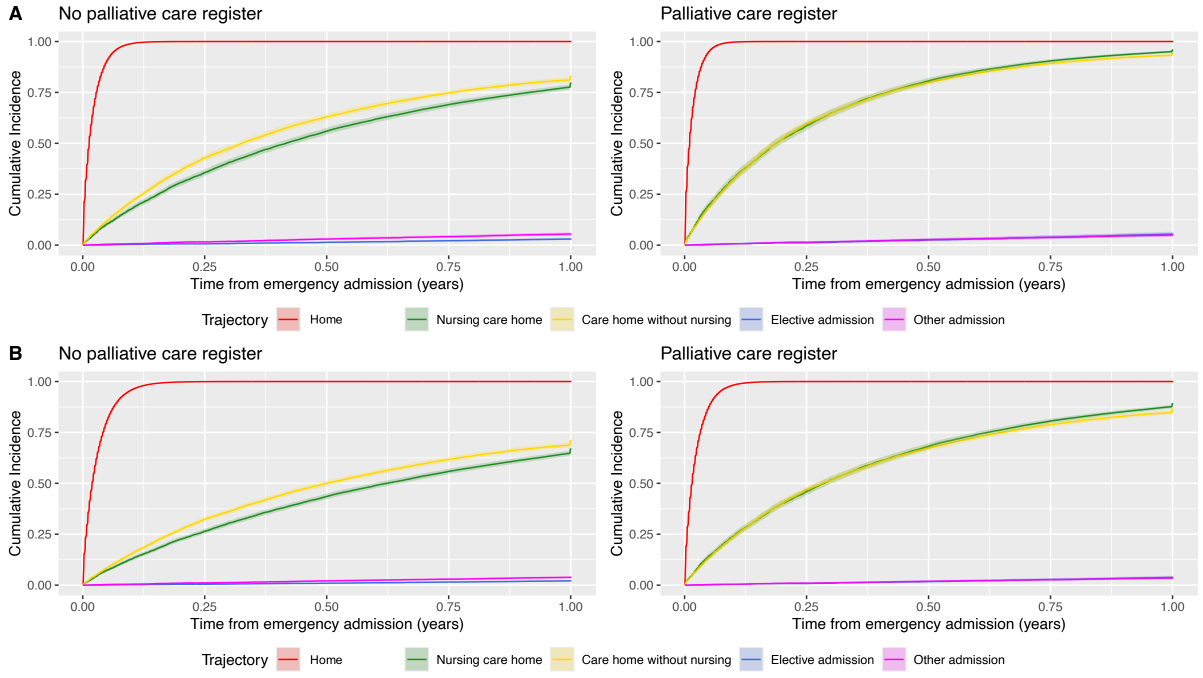

A: 25 years; B: 50 years

v) Transitions from elective hospital admission

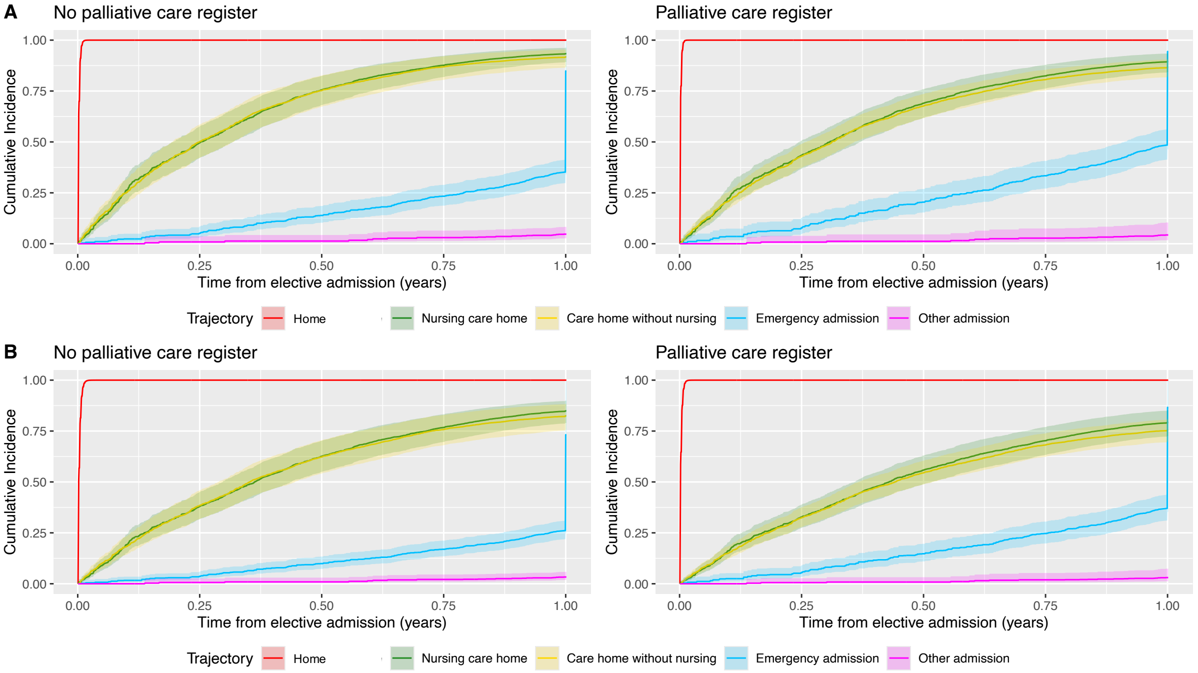

A: 25 years; B: 50 years

Supplementary Figure 7: Cumulative incidence plots for females adjusted for age, area-level deprivation, rurality, and palliative care register

i) Transitions from home

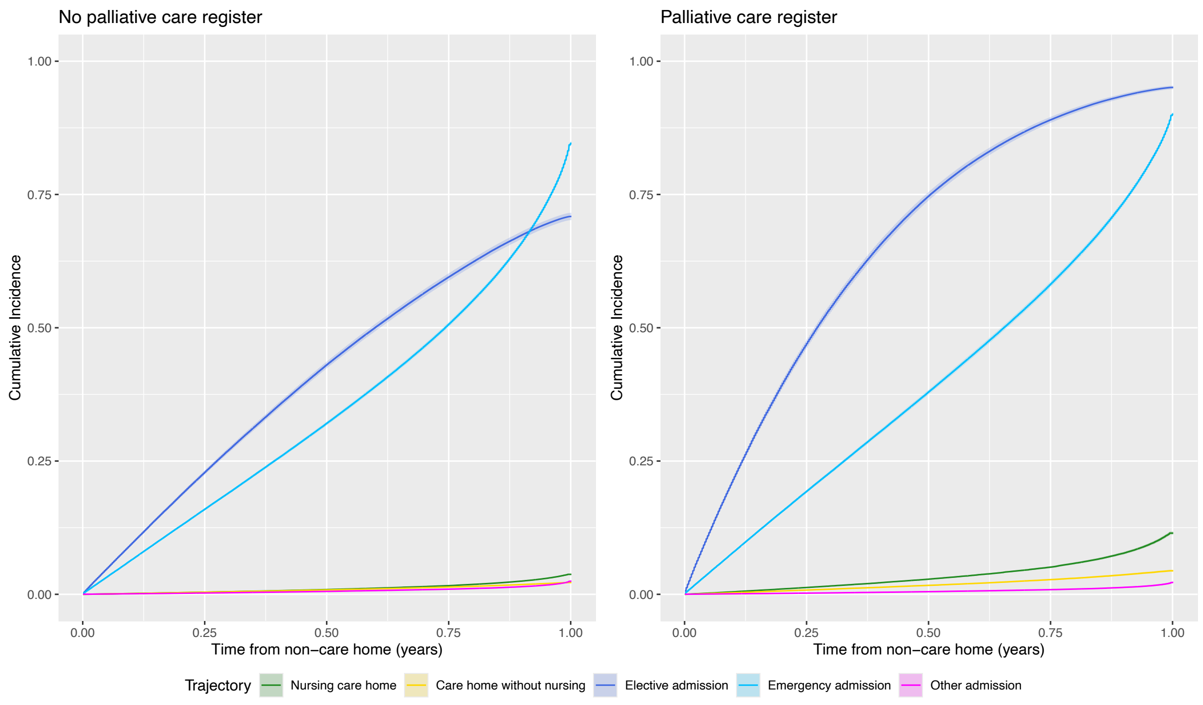

ii) Transitions from nursing care home

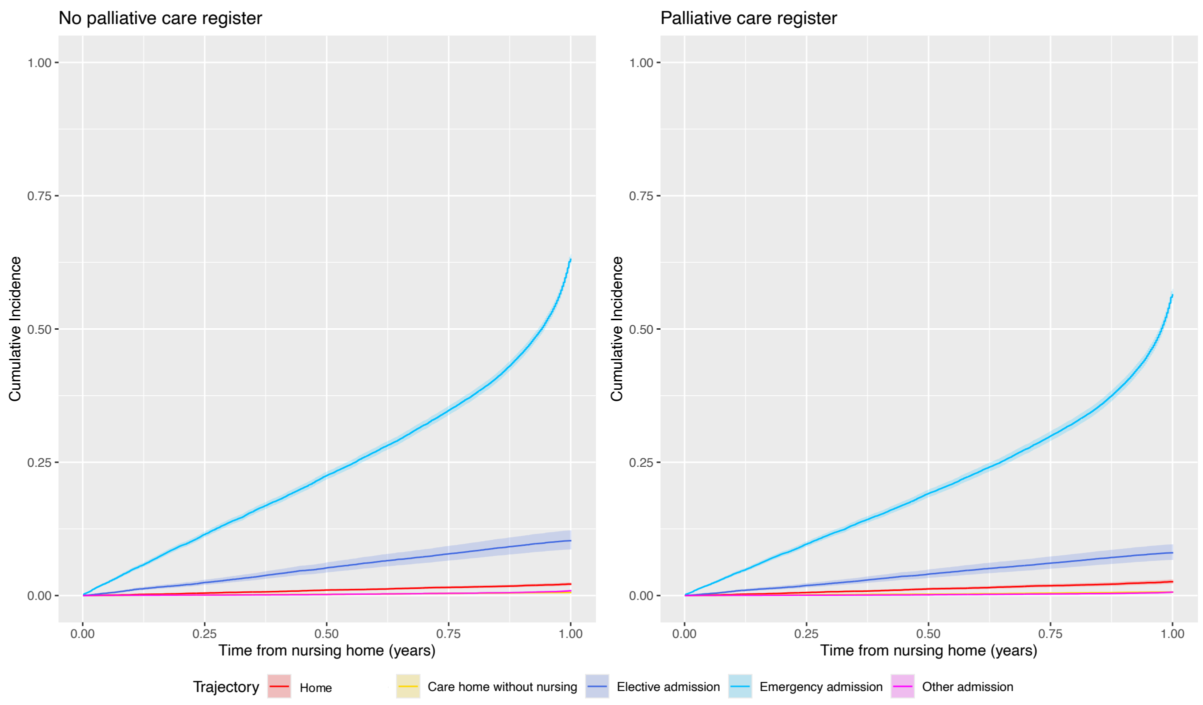

iii) Transitions from care home without nursing

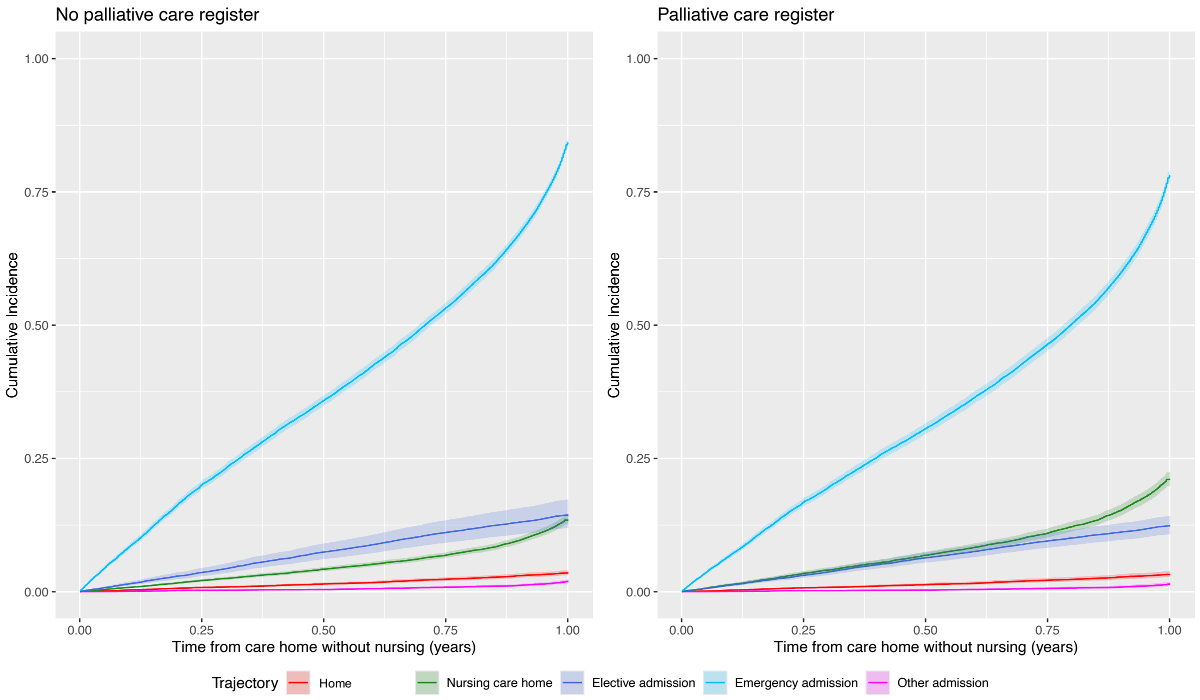

iv) Transitions from emergency hospital admission

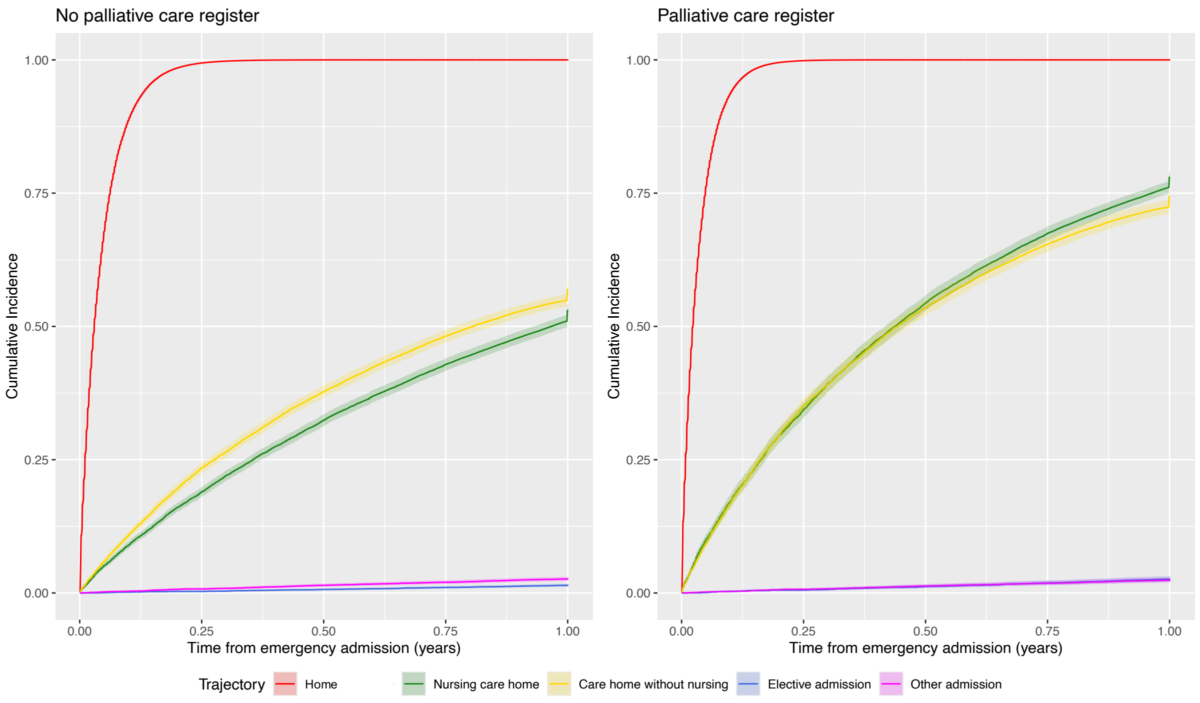

v) Transitions from elective hospital admission

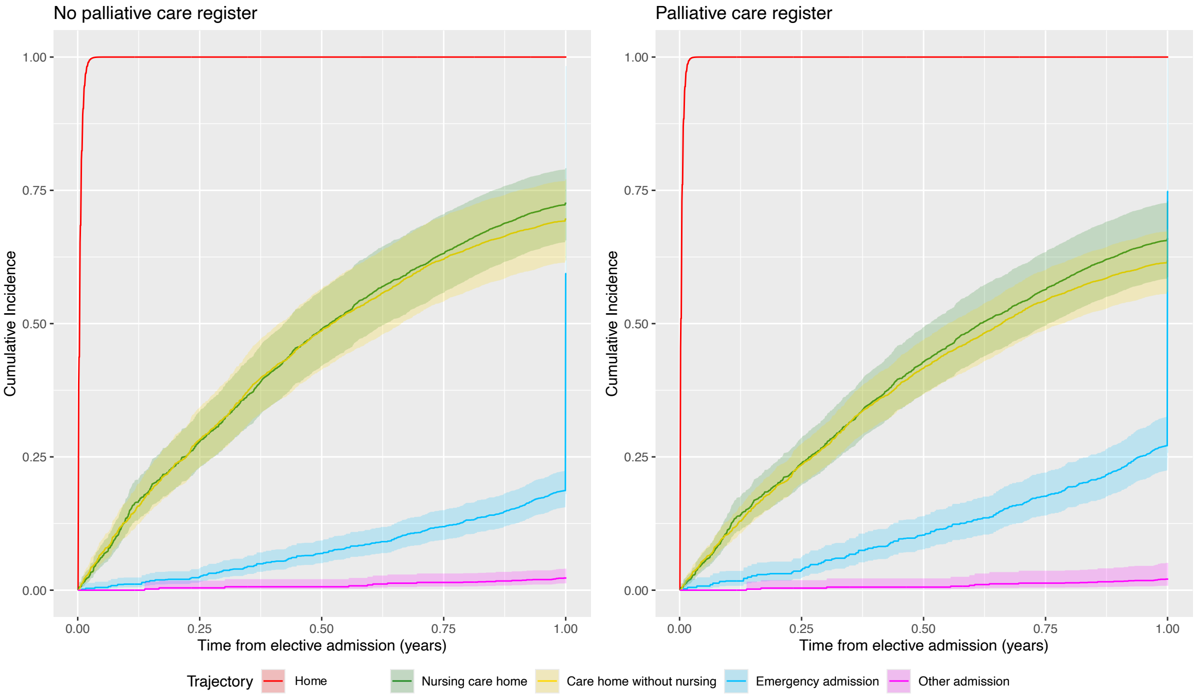

Supplementary Figure 8: Cumulative incidence plots for least deprived adjusted for age, sex, rurality, and palliative care register

i) Transitions from home

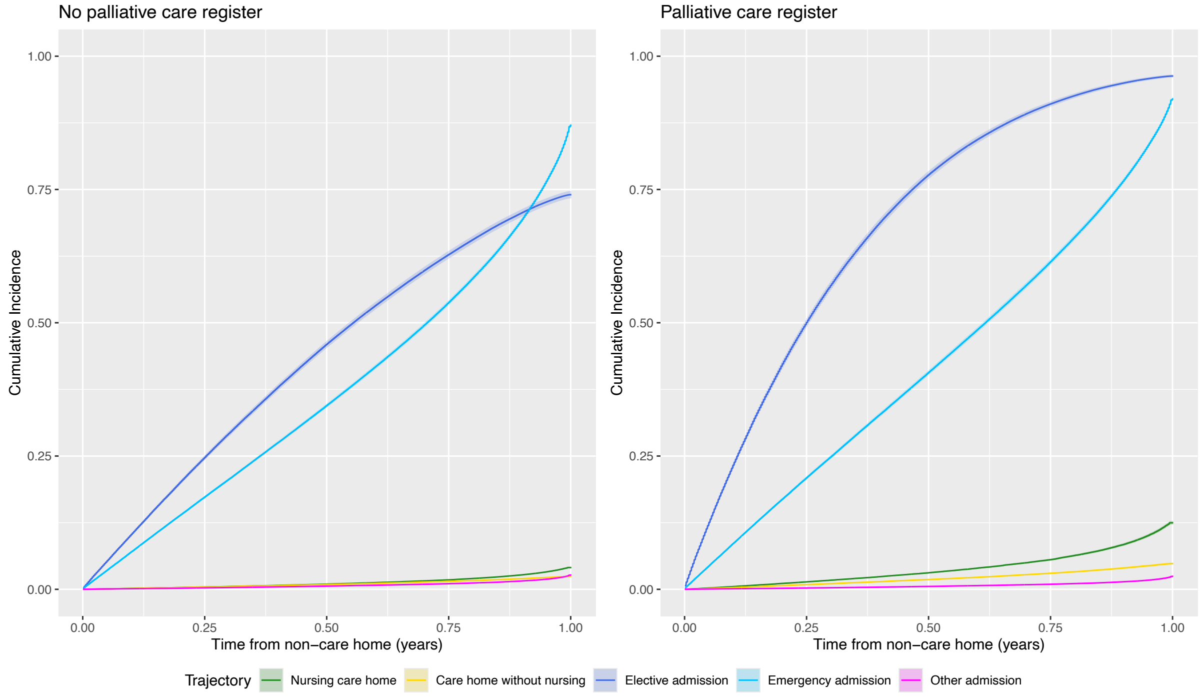

ii) Transitions from nursing care home

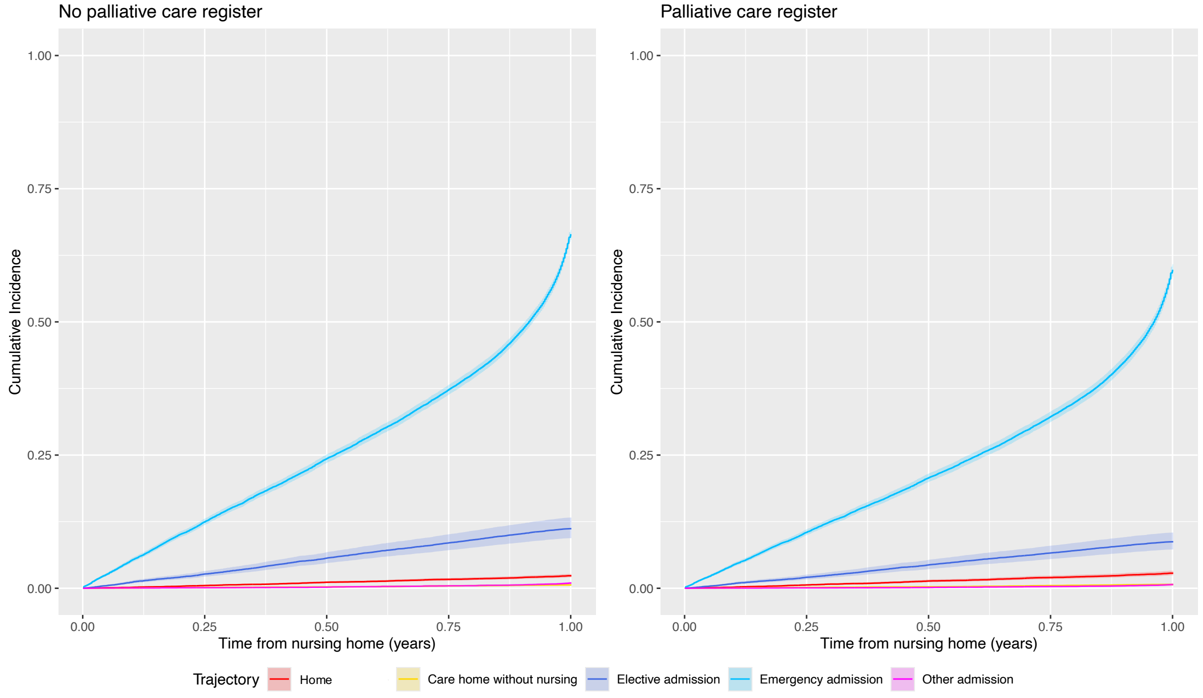

iii) Transitions from care home without nursing

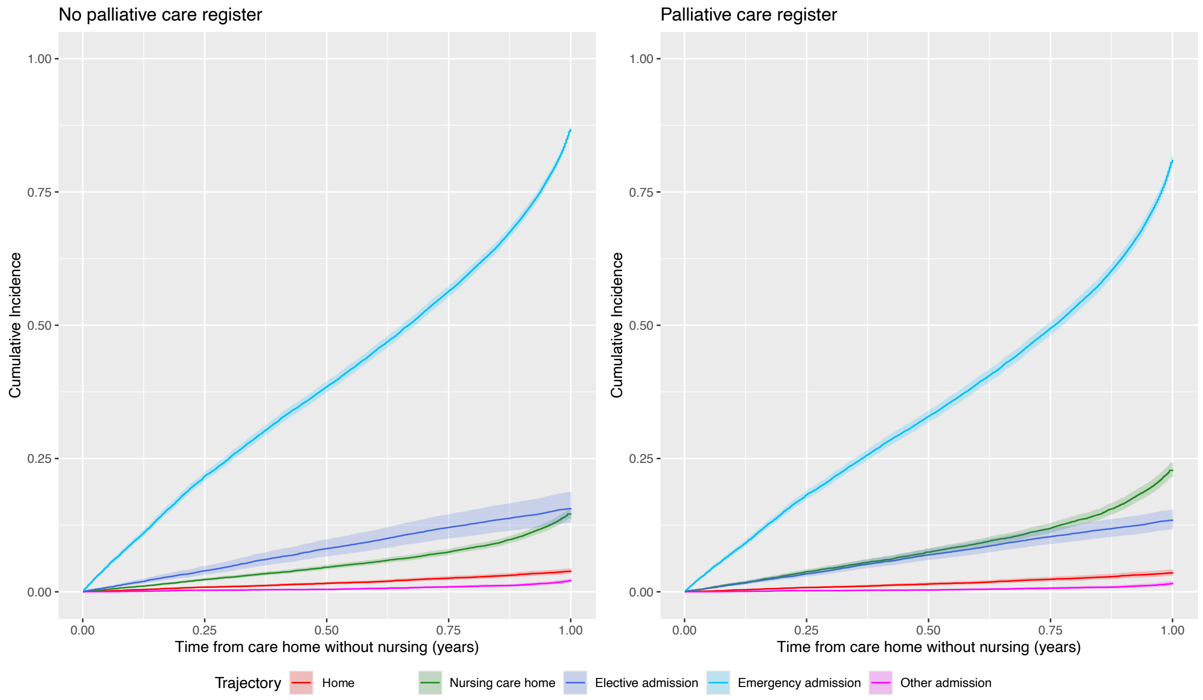

iv) Transitions from emergency hospital admission

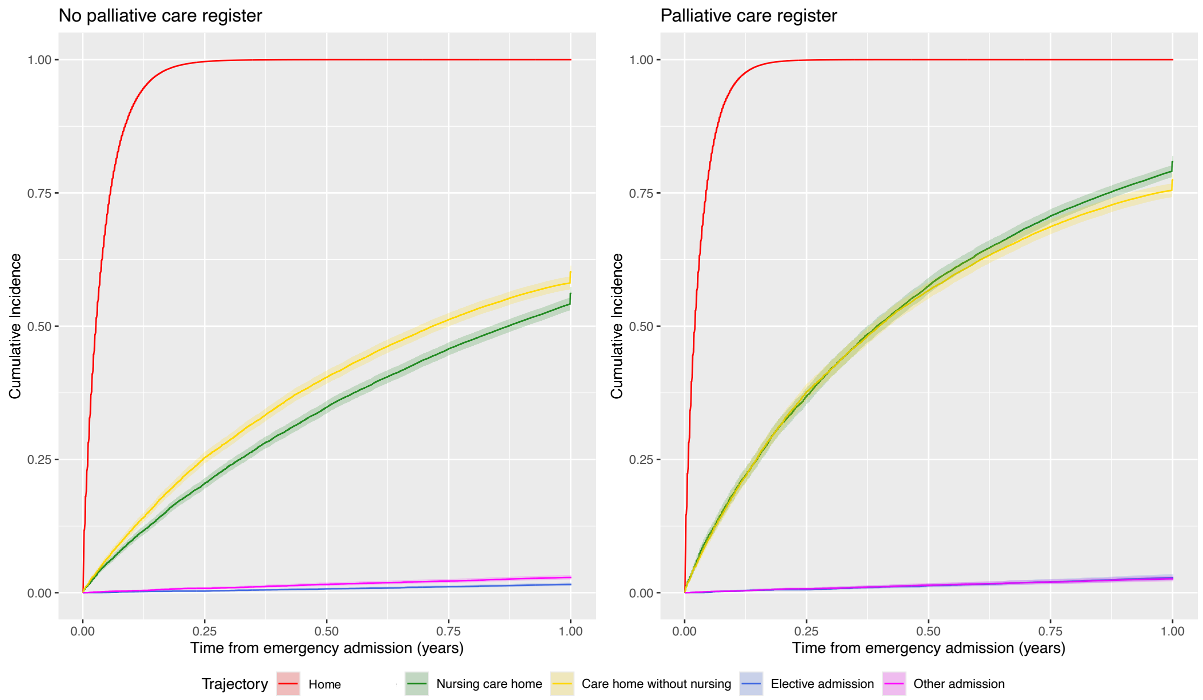

v) Transitions from elective hospital admission

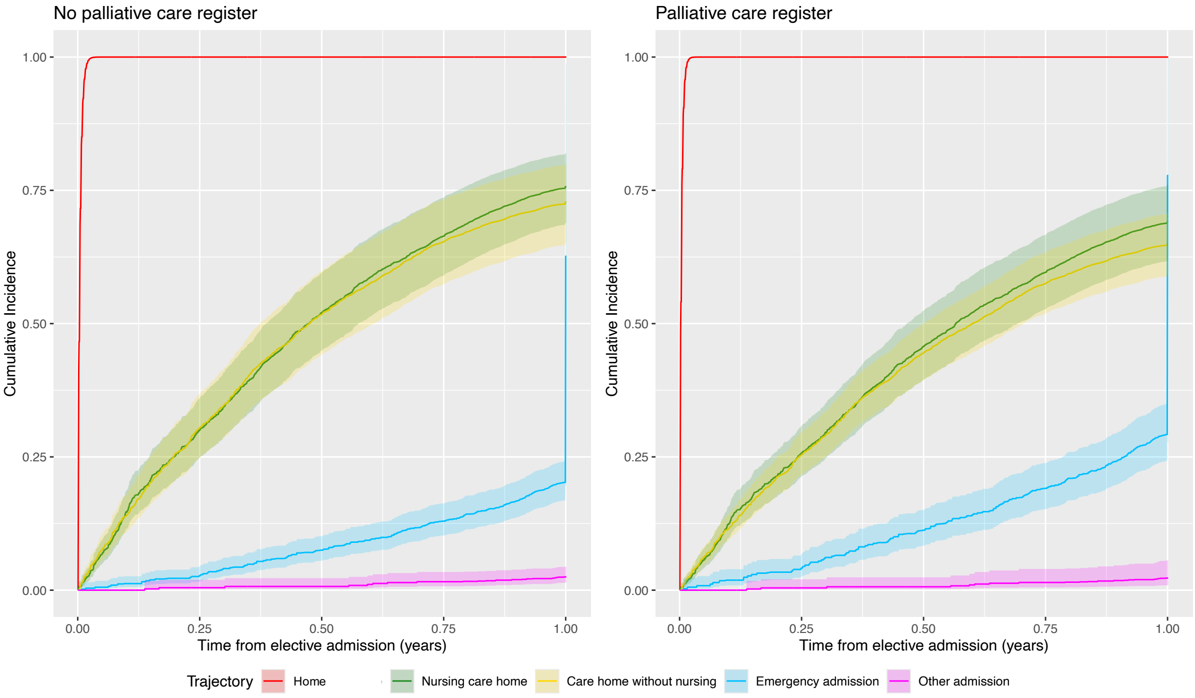

Supplementary Figure 9: Cumulative incidence plots for rural areas adjusted for age, sex, area-level deprivation, and palliative care register

i) Transitions from home

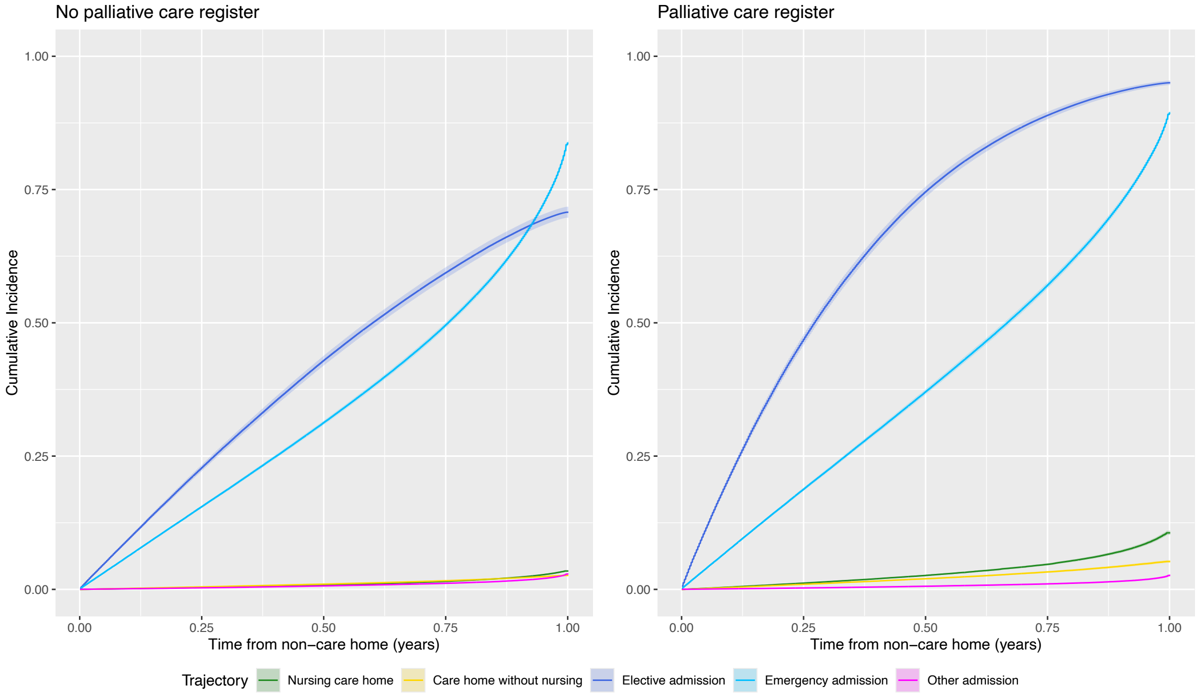

ii) Transitions from nursing care home

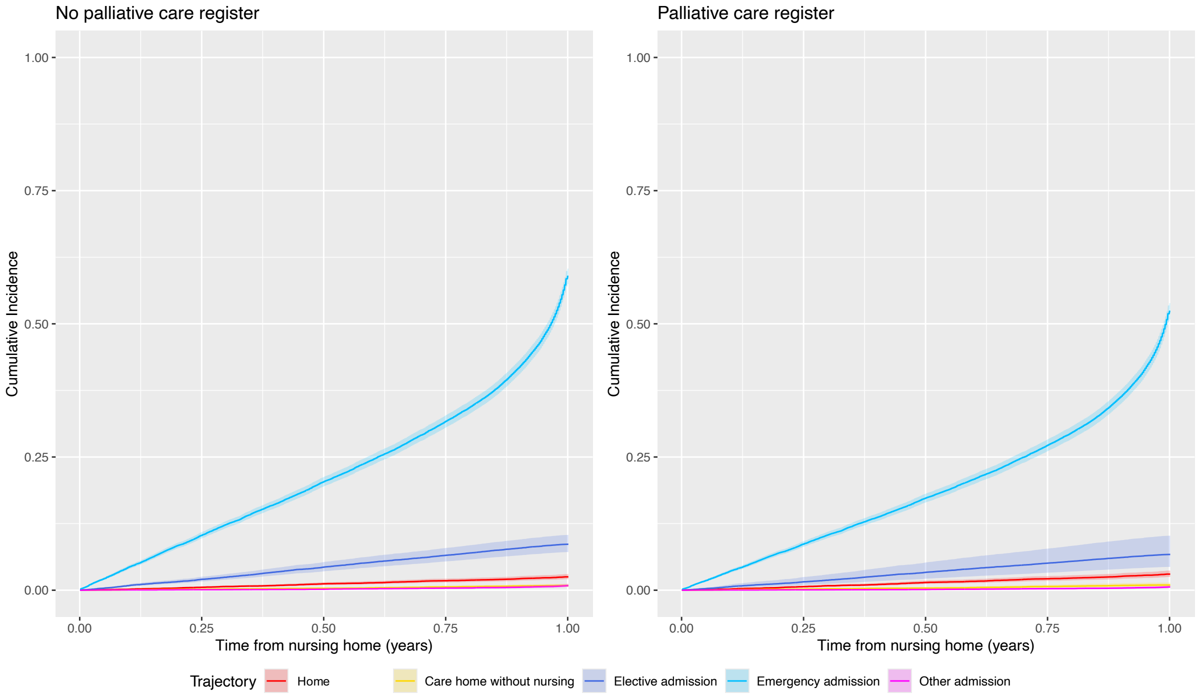

iii) Transitions from care home without nursing

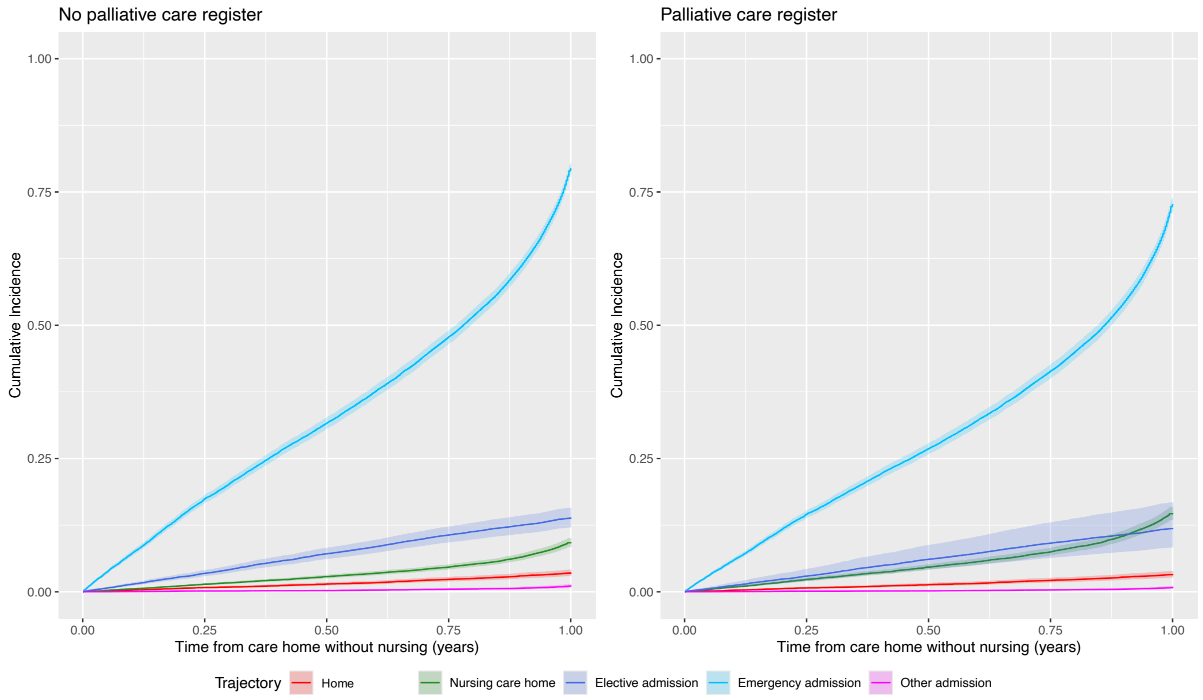

iv) Transitions from emergency hospital admission

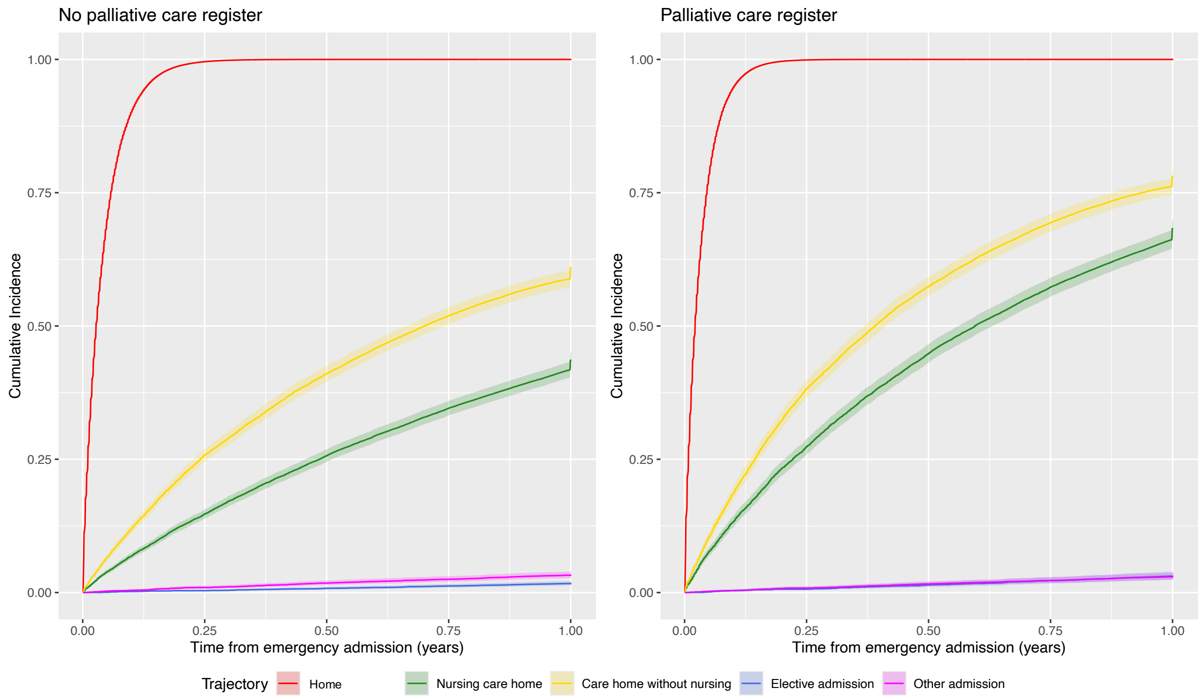

v) Transitions from elective hospital admission

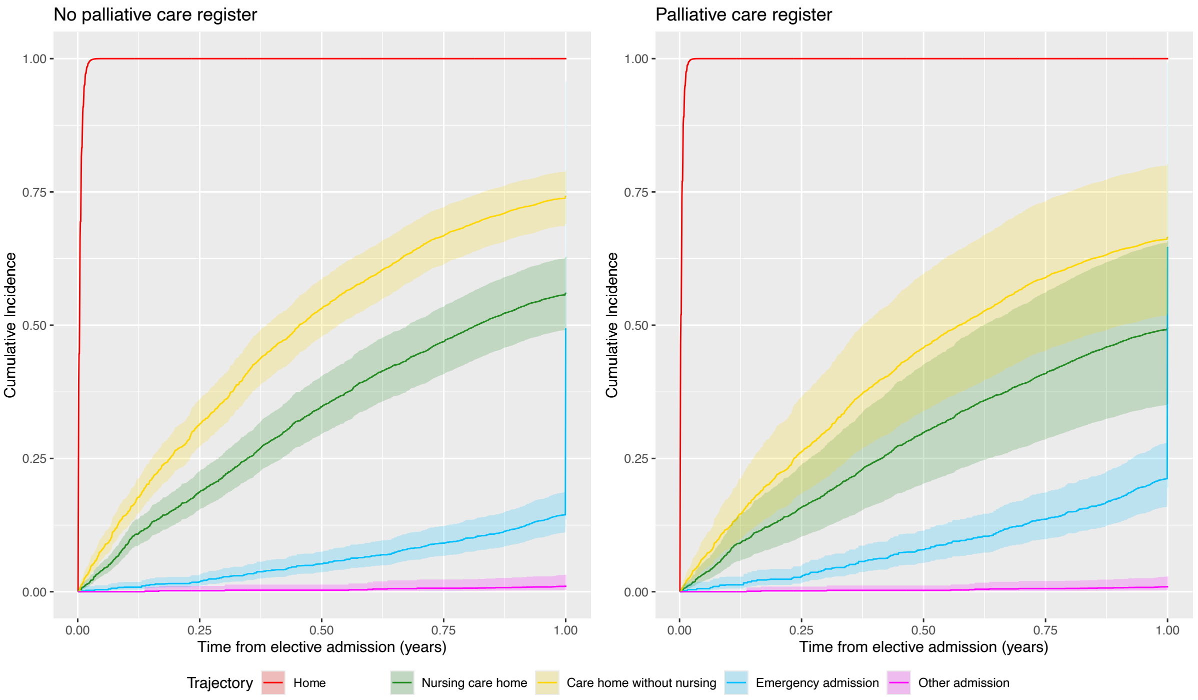

Supplementary Figure 10: Sensitivity analysis - cumulative incidence plots for frailty adjusted for age, sex, rurality, frailty status, and area-level deprivation as fixed covariates, and palliative care register as transition specific covariates

i) Transitions from home

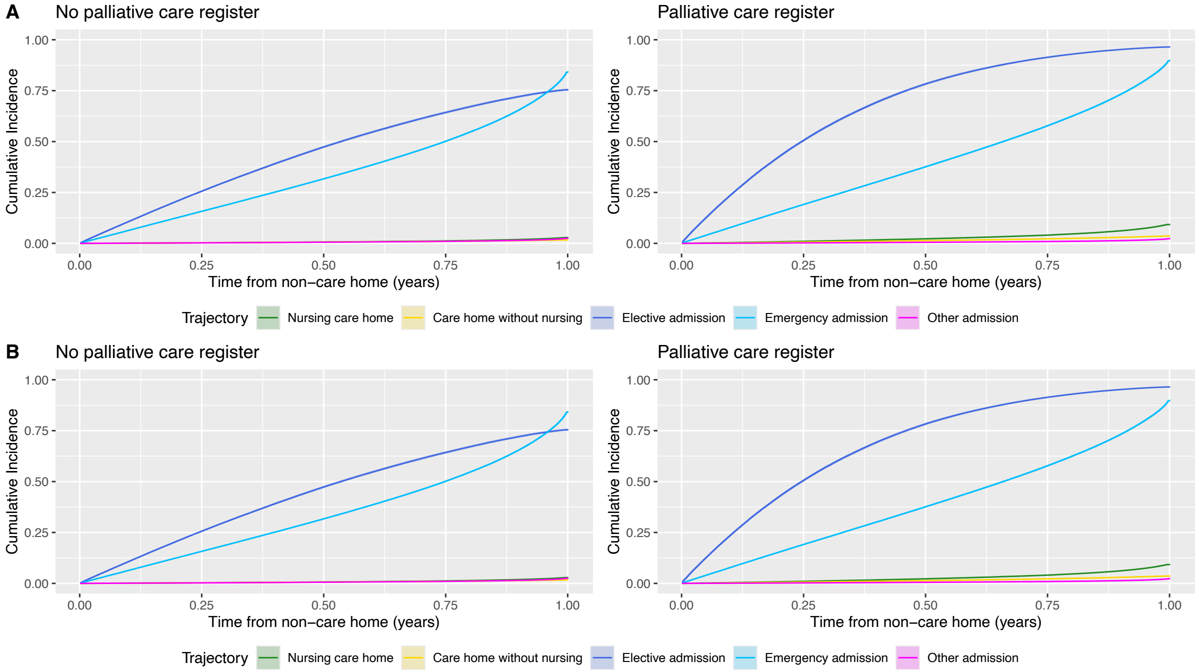

A: Severe; B: Fit

ii) Transitions from nursing care home

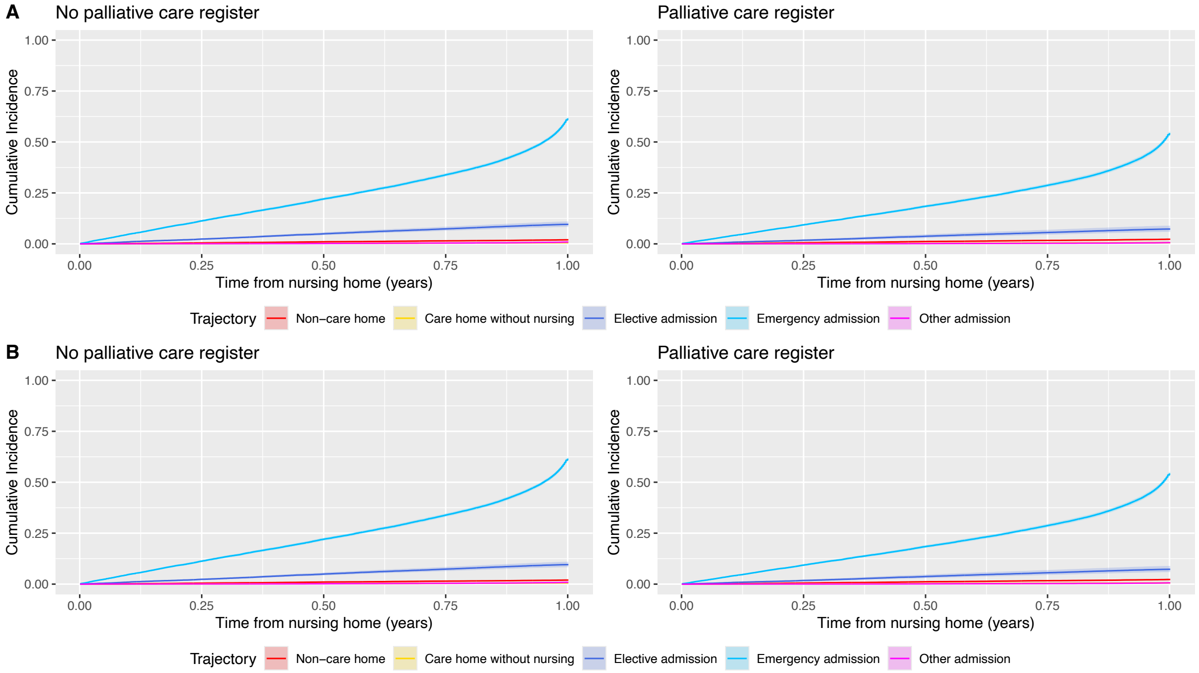

A: Severe; B: Fit

iii) Transitions from care home without nursing

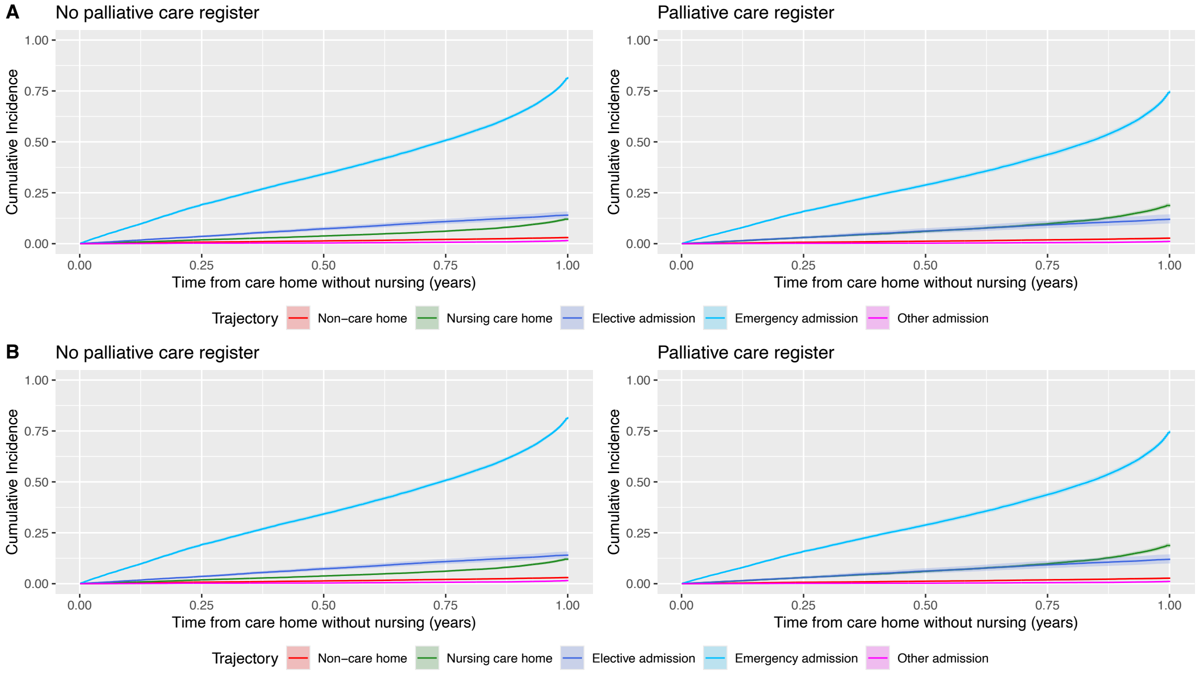

A: Severe; B: Fit

iv) Transitions from emergency hospital admission

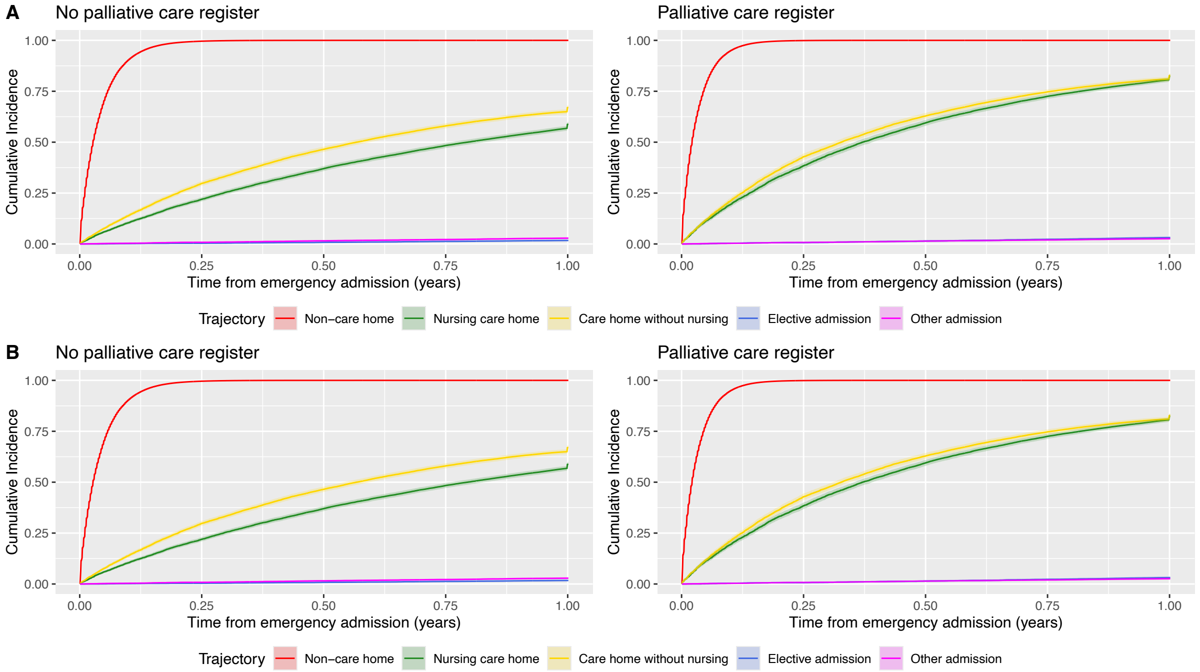

A: Severe; B: Fit

v) Transitions from elective hospital admission

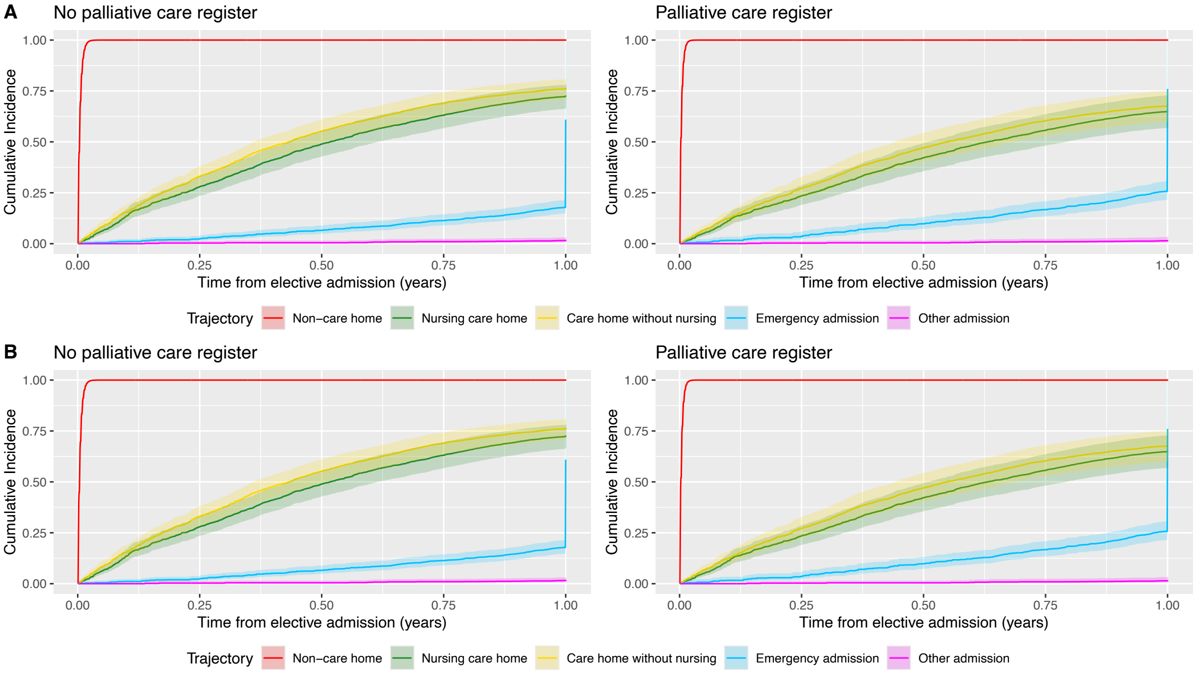

A: Severe; B: Fit

Supplementary Figure 11: Sensitivity analysis - cumulative incidence plots for frailty assuming worst case (all missing data are severe) and best case (all missing data are fit) adjusted for age, sex, rurality, and area-level deprivation as fixed covariates, and palliative care register as transition specific covariates

i) Transitions from home

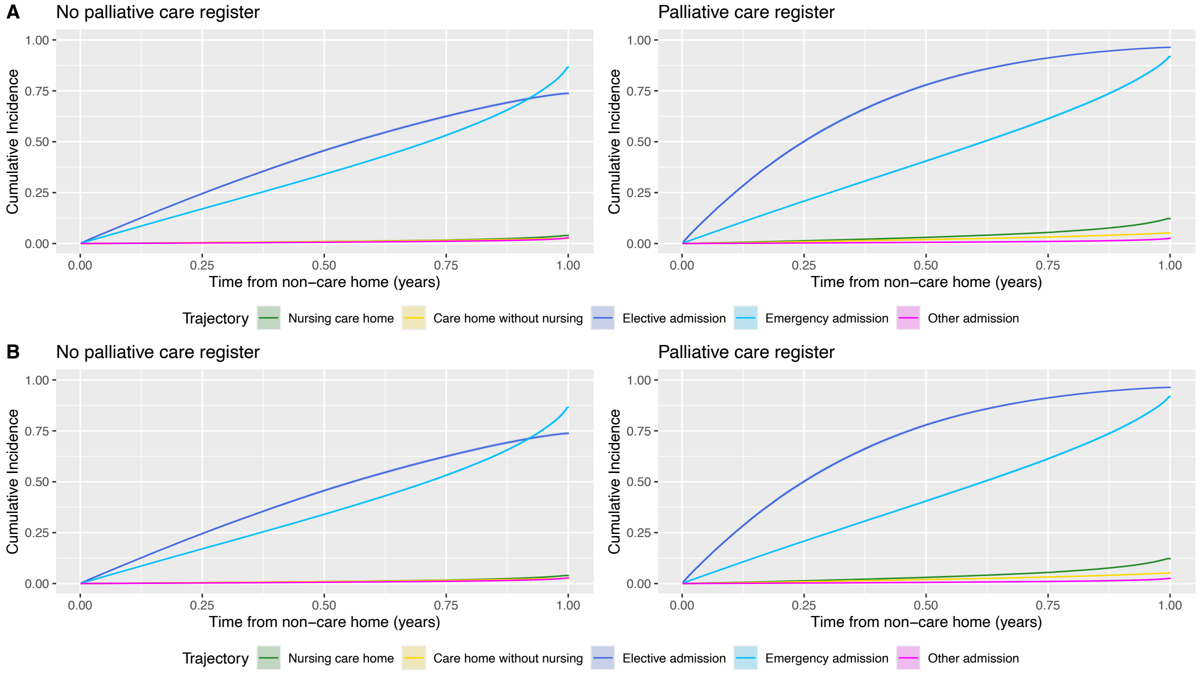

A: Severe; B: Fit

ii) Transitions from nursing care home

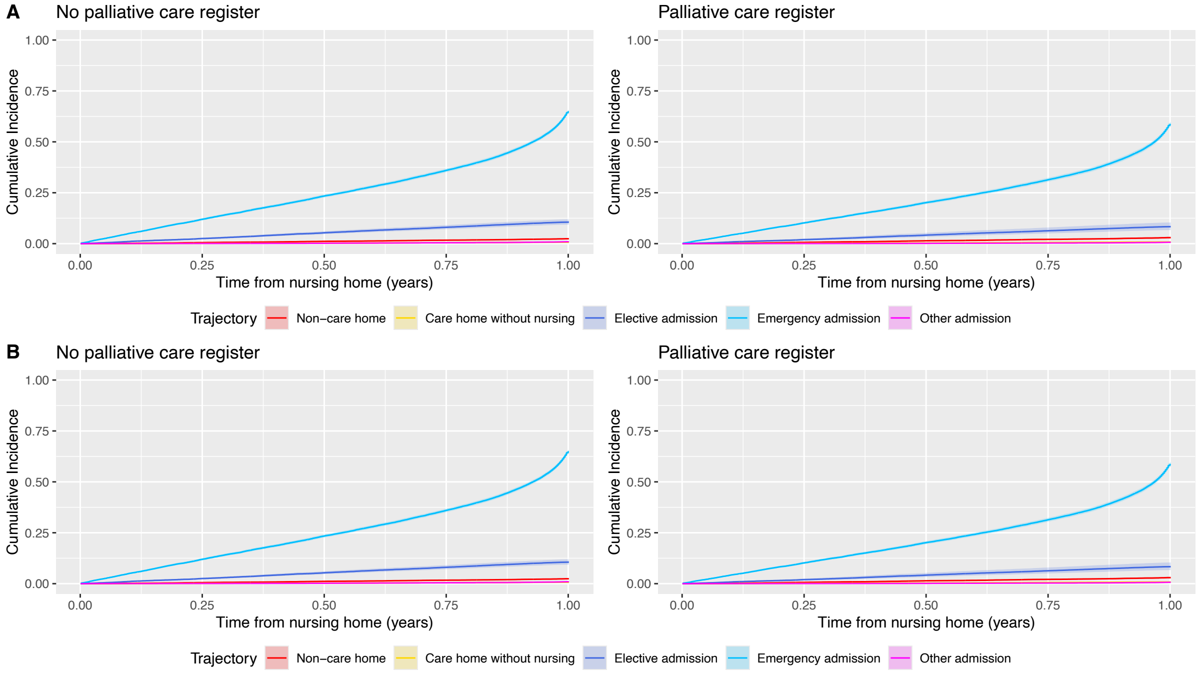

A: Severe; B: Fit

iii) Transitions from care home without nursing

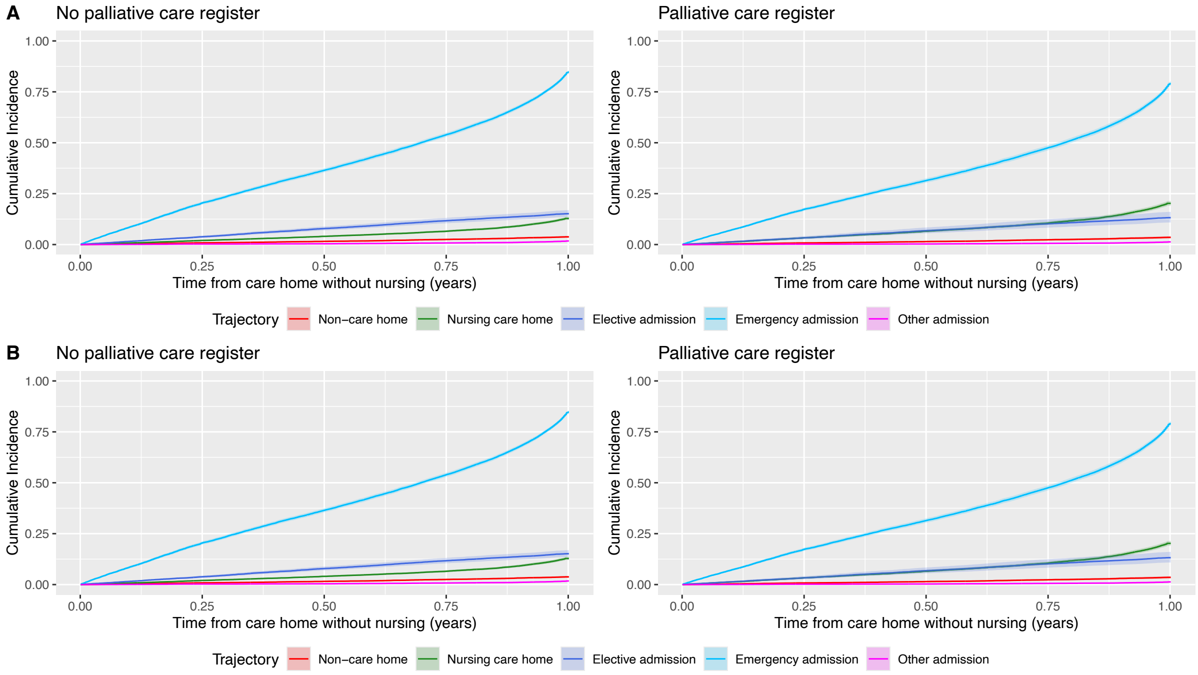

A: Severe; B: Fit

iv) Transitions from emergency hospital admission

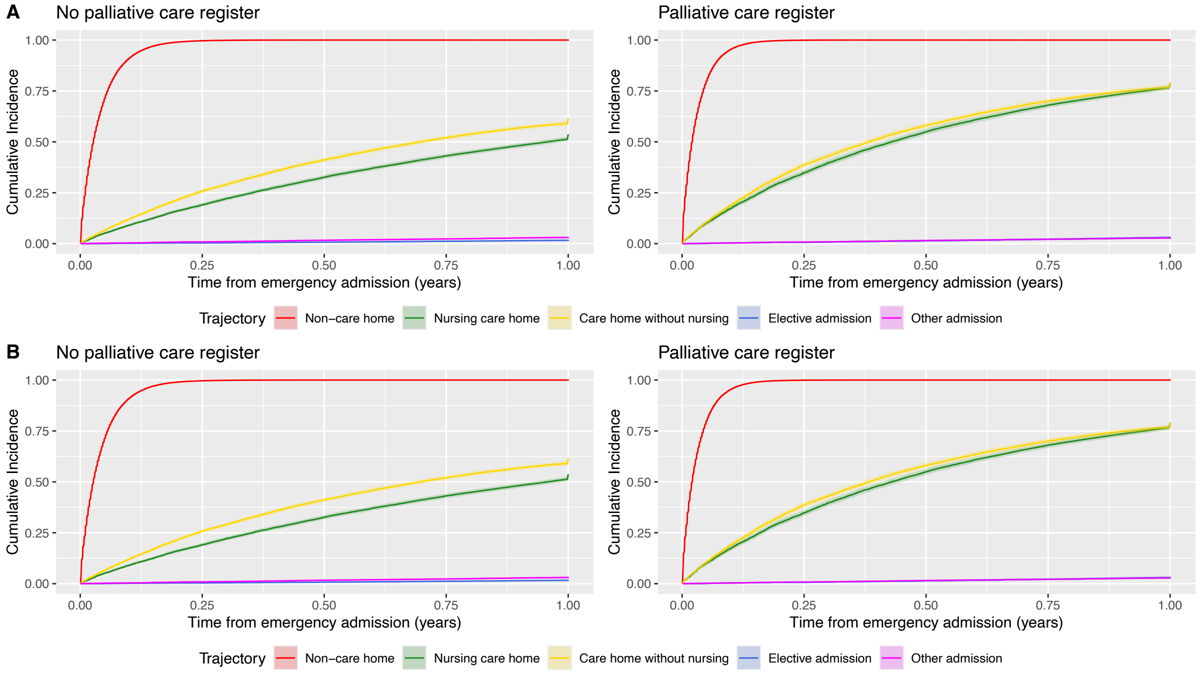

A: Severe; B: Fit

v) Transitions from elective hospital admission

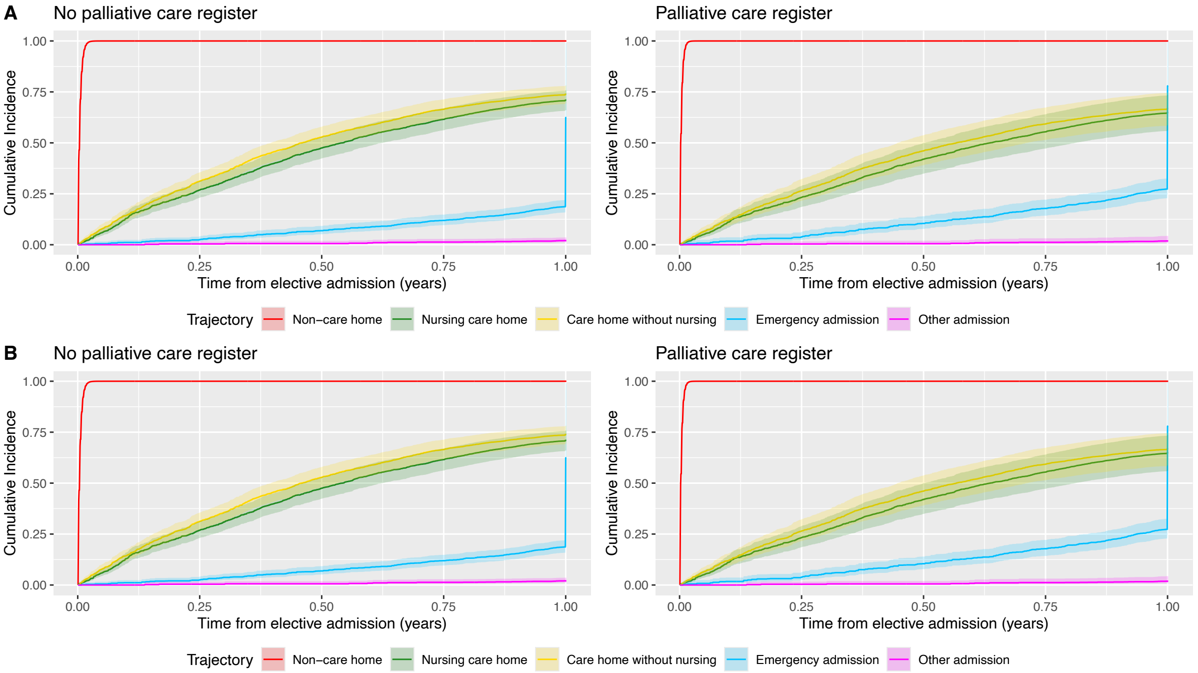

A: Severe; B: Fit

Supplementary Figure 12: Sensitivity analysis - cumulative incidence plots for living alone, adjusted for age, sex, rurality, and area-level deprivation as fixed covariates, and palliative care register and living alone as transition specific covariates

i) Transitions from home

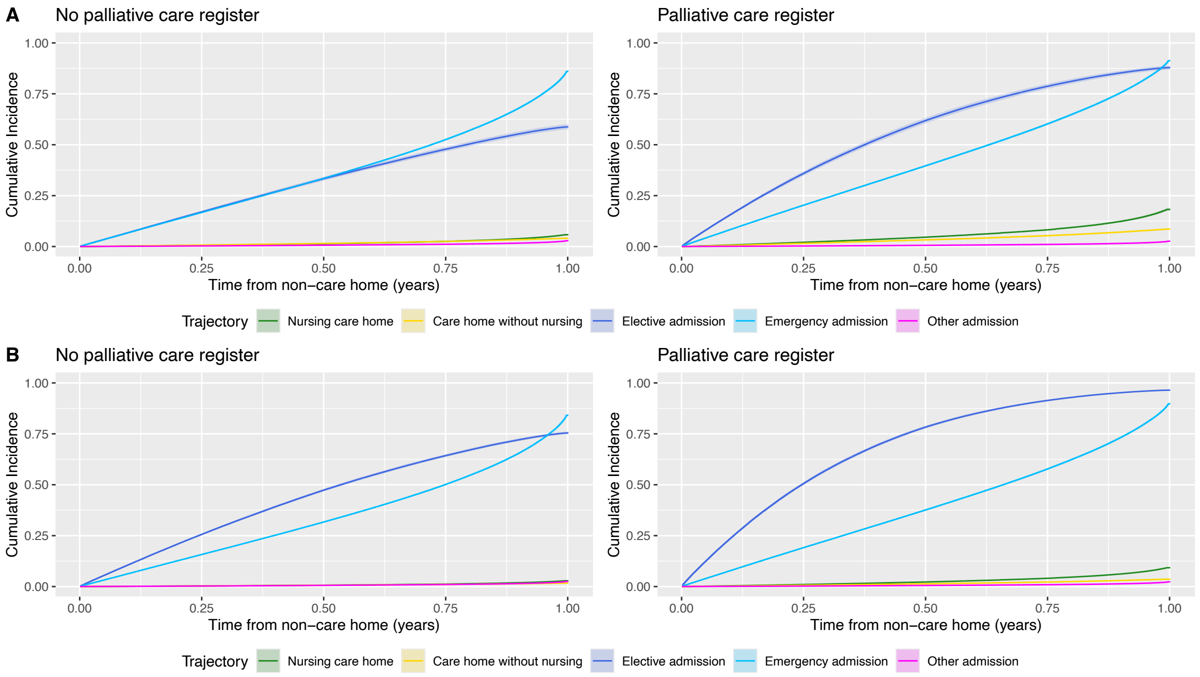

A: Alone; B: Not alone

ii) Transitions from nursing care home

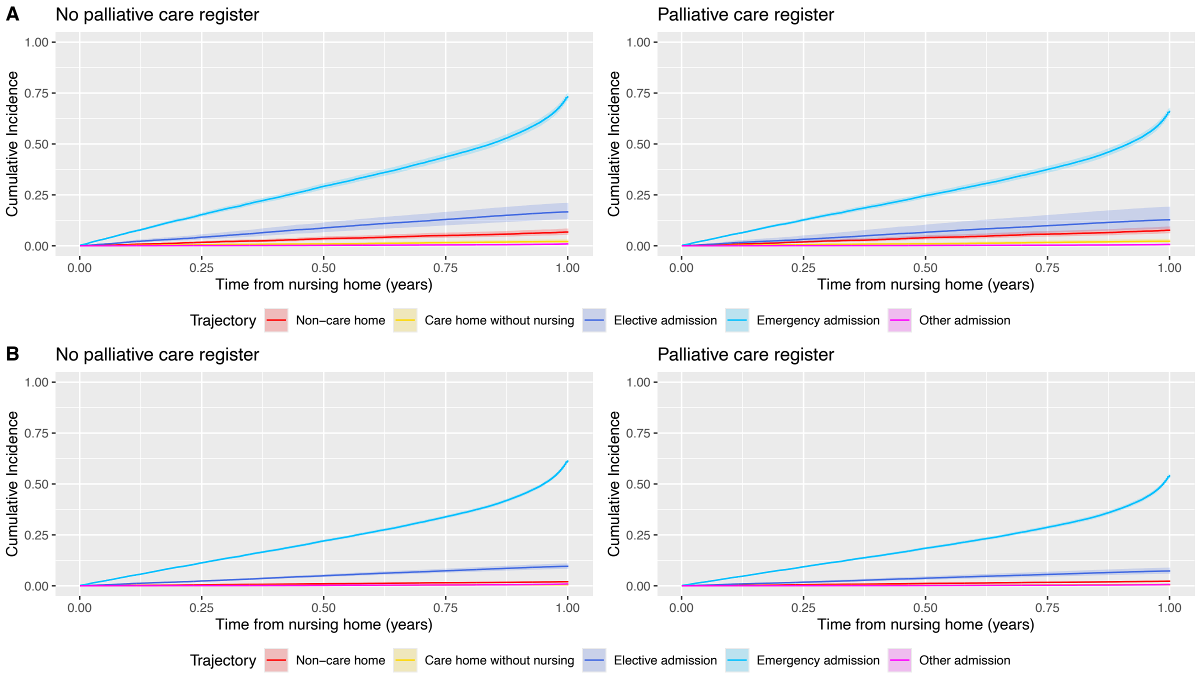

A: Alone; B: Not alone

iii) Transitions from care home without nursing

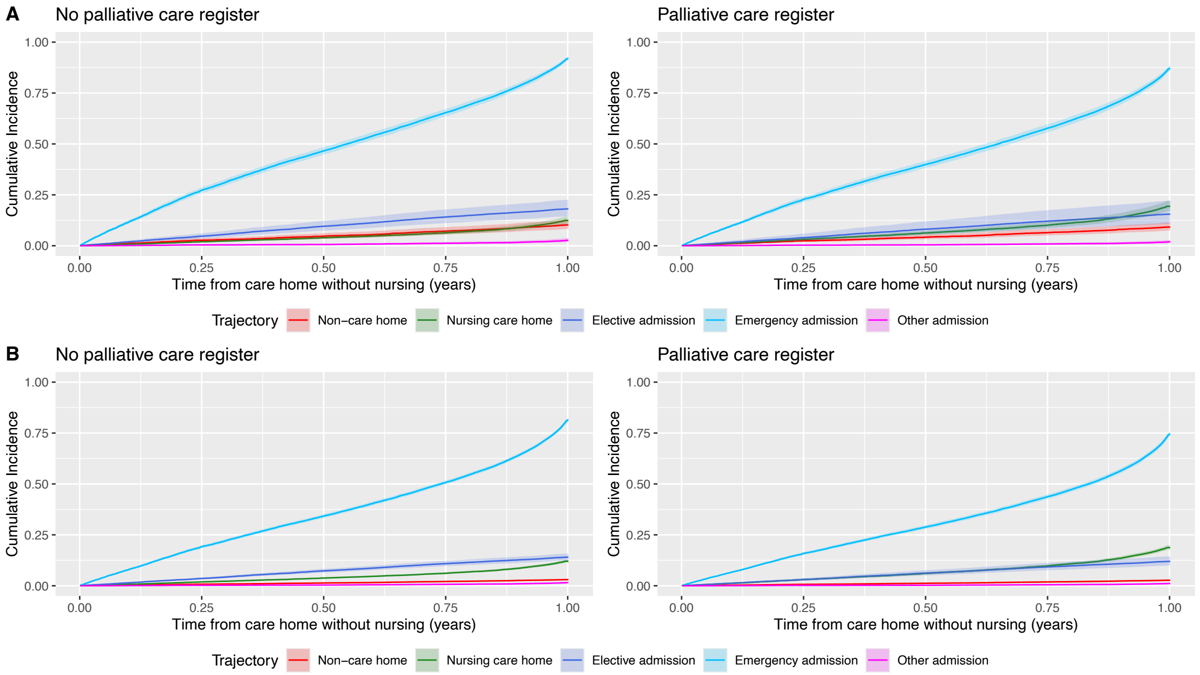

A: Alone; B: Not alone

iv) Transitions from emergency hospital admission

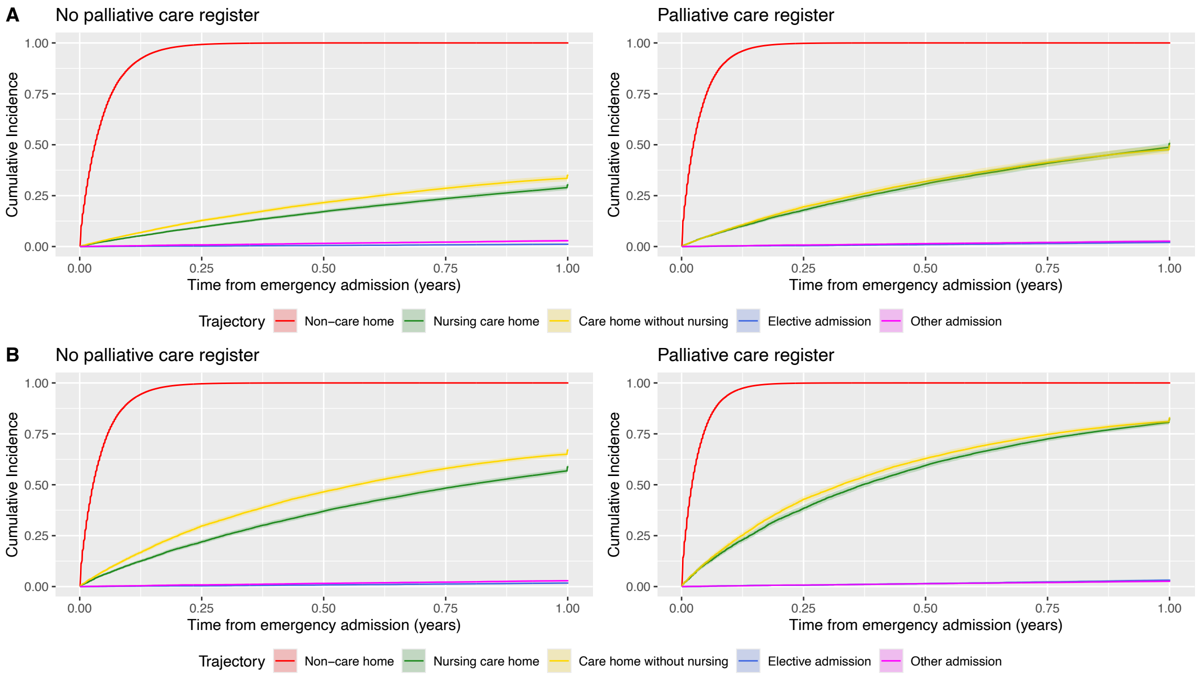

A: Alone; B: Not alone

v) Transitions from elective hospital admission

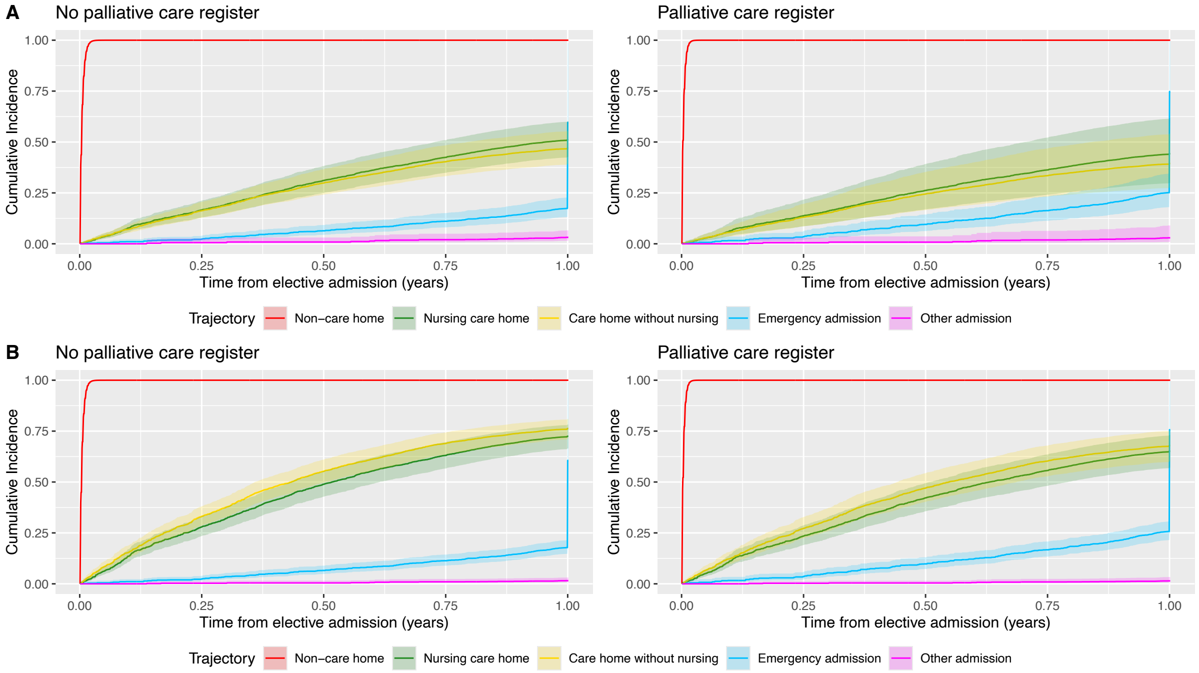

A: Alone; B: Not alone

Supplementary Figure 13: Sensitivity analysis - cumulative incidence plots for palliative care registration within 6 months of death, adjusted for age, sex, and area-level deprivation as fixed covariates, and rurality and palliative care register as transition specific covariates

i) Transitions from home

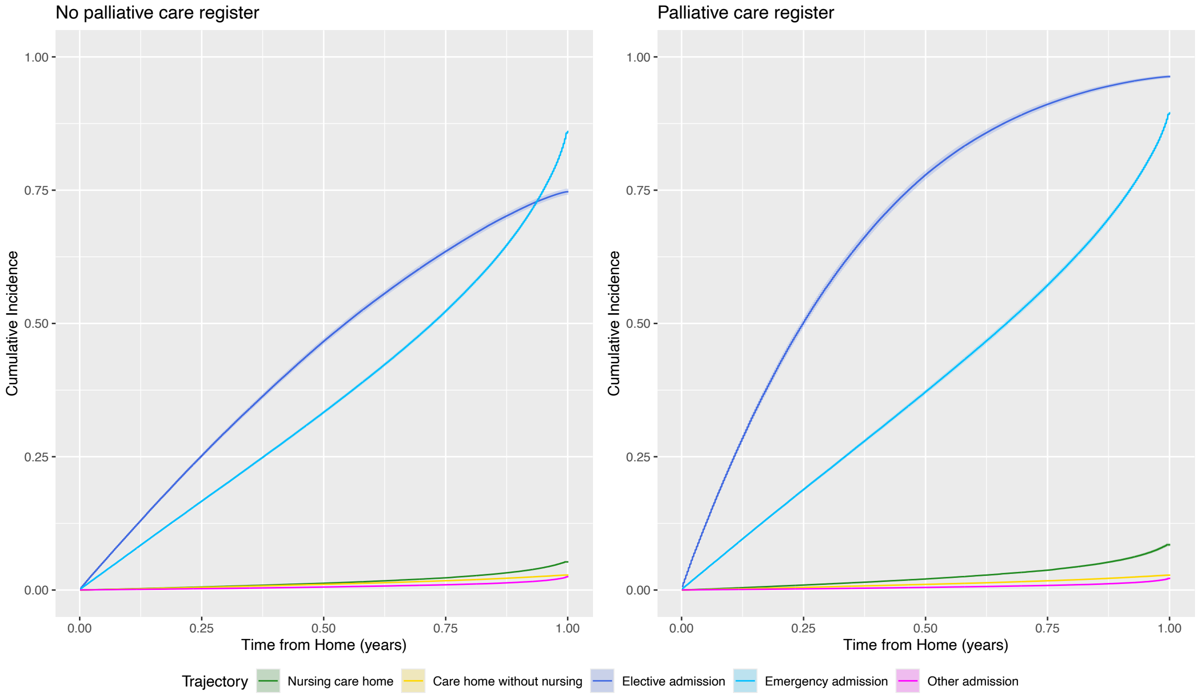

ii) Transitions from nursing care home

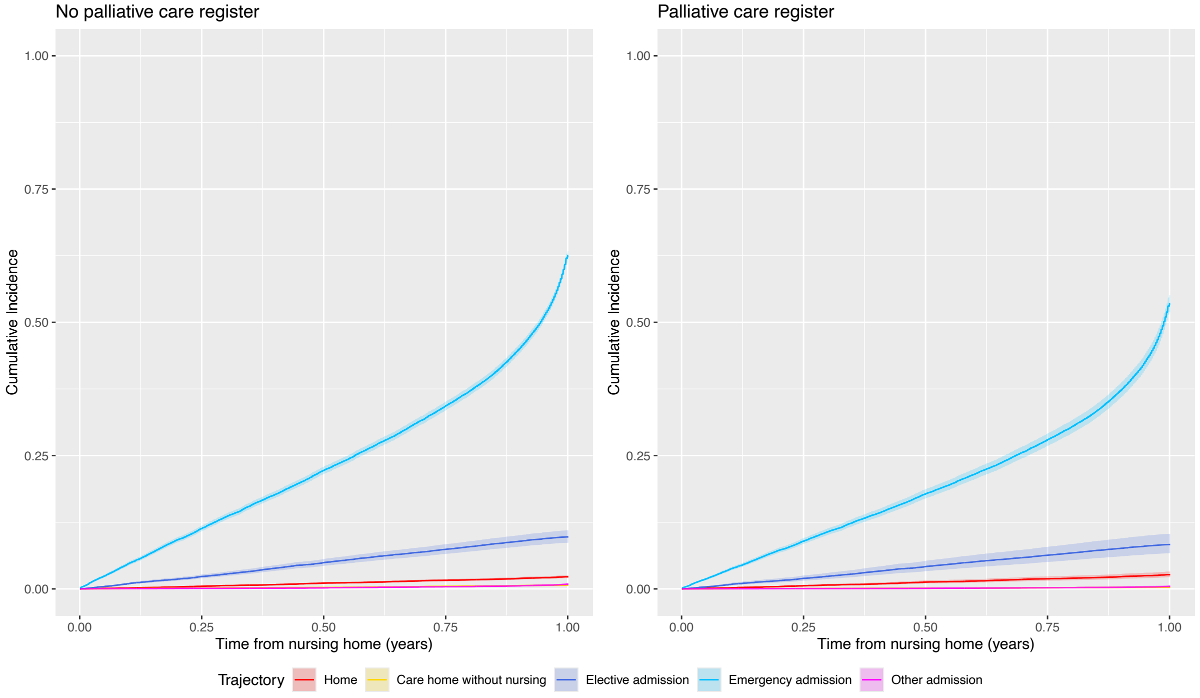

iii) Transitions from care home without nursing

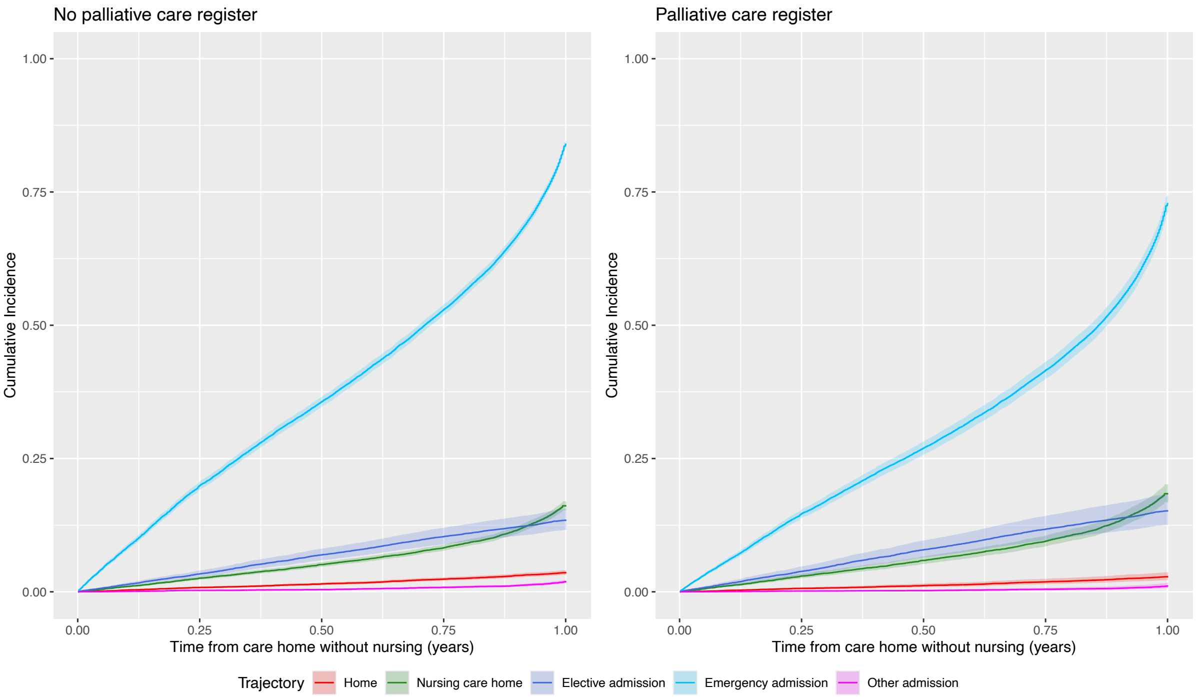

iv) Transitions from emergency hospital admission

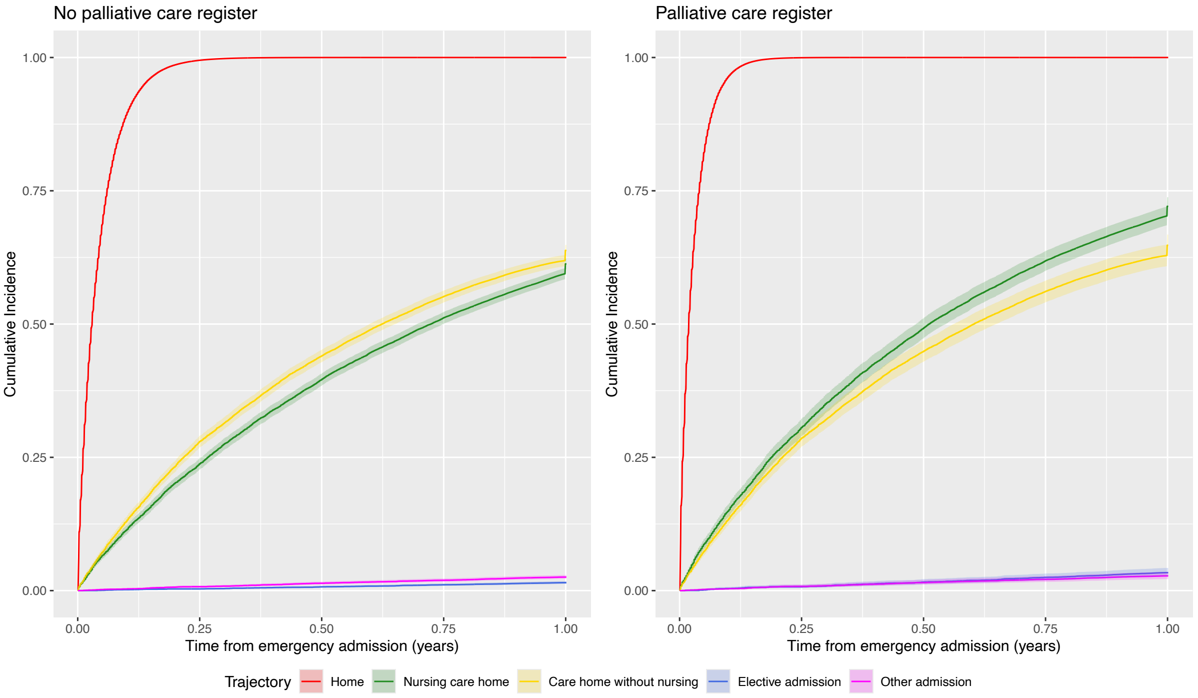

v) Transitions from elective hospital admission

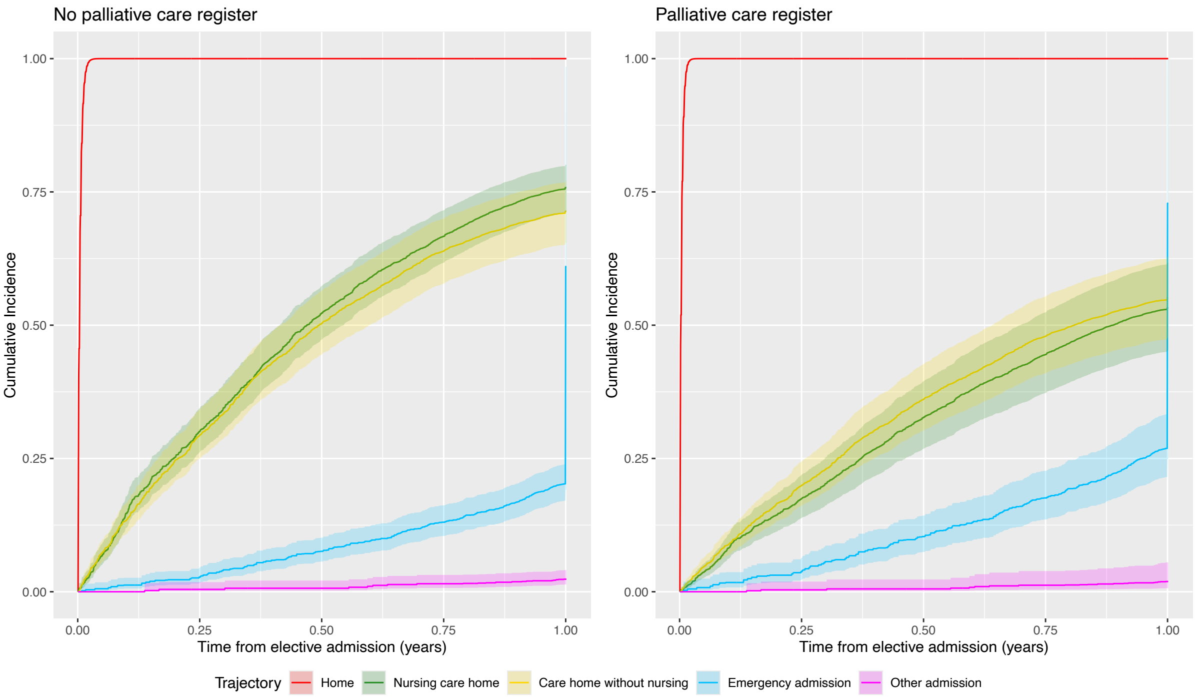

Supplementary Figure 14: Sensitivity analysis - cumulative incidence plots for palliative care registration within 1 month of death, adjusted for age, sex, and area-level deprivation as fixed covariates, and rurality and palliative care register as transition specific covariates

i) Transitions from home

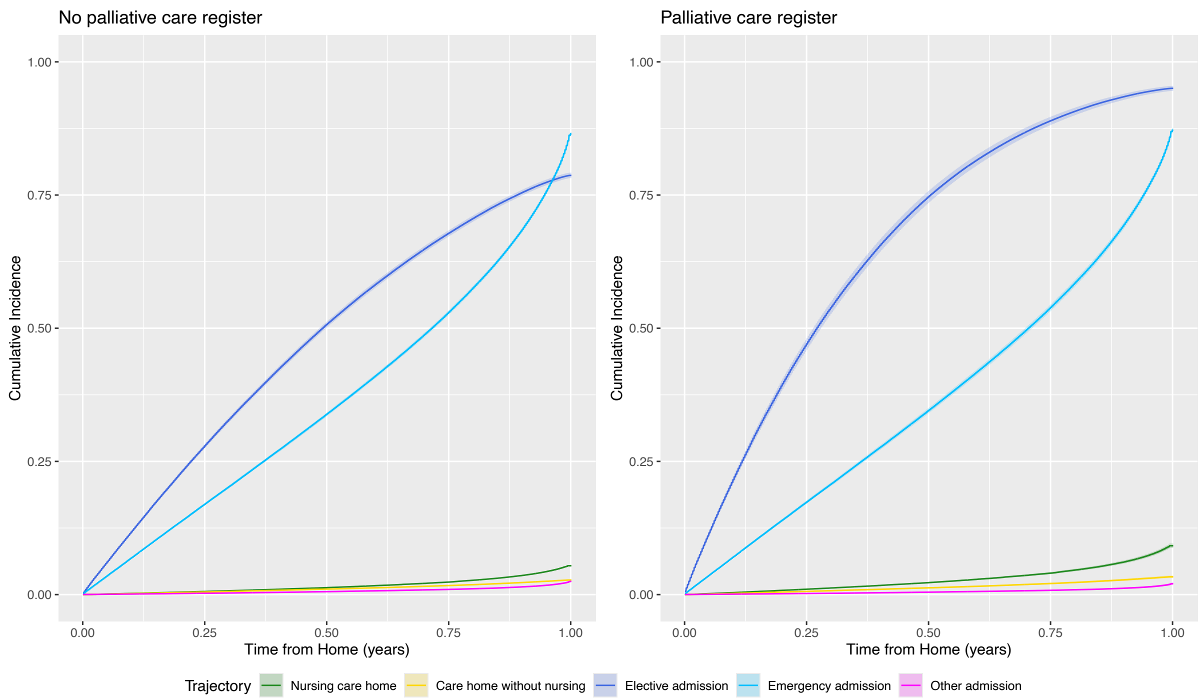

ii) Transitions from nursing care home

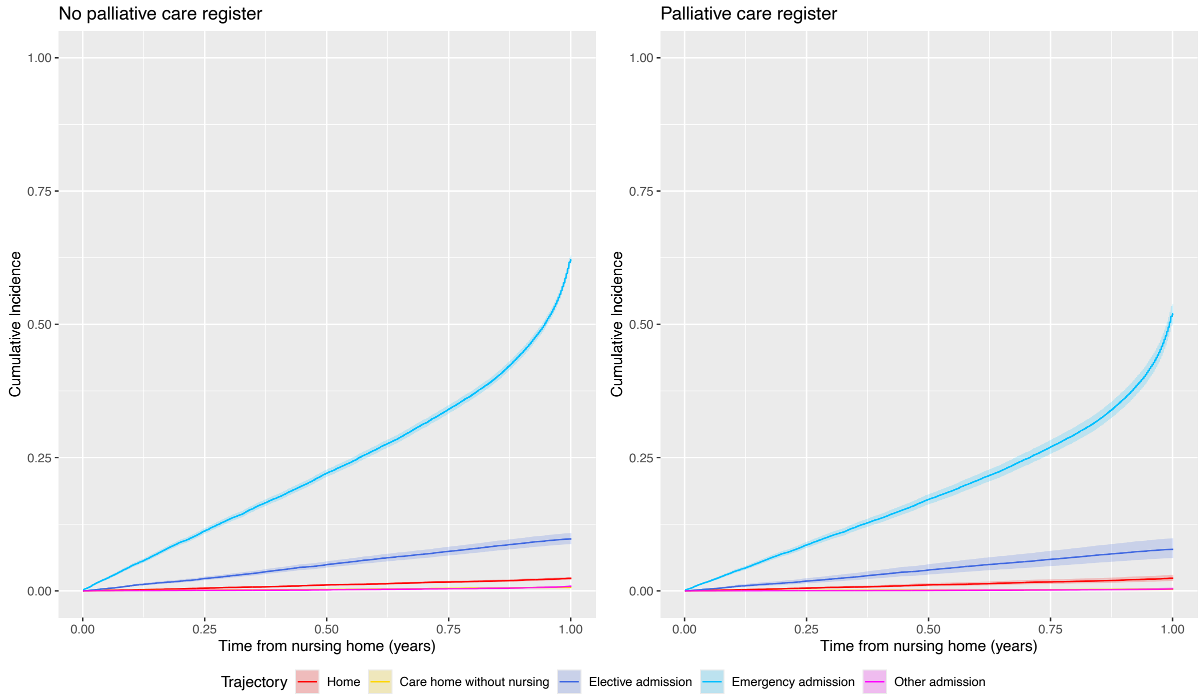

iii) Transitions from care home without nursing

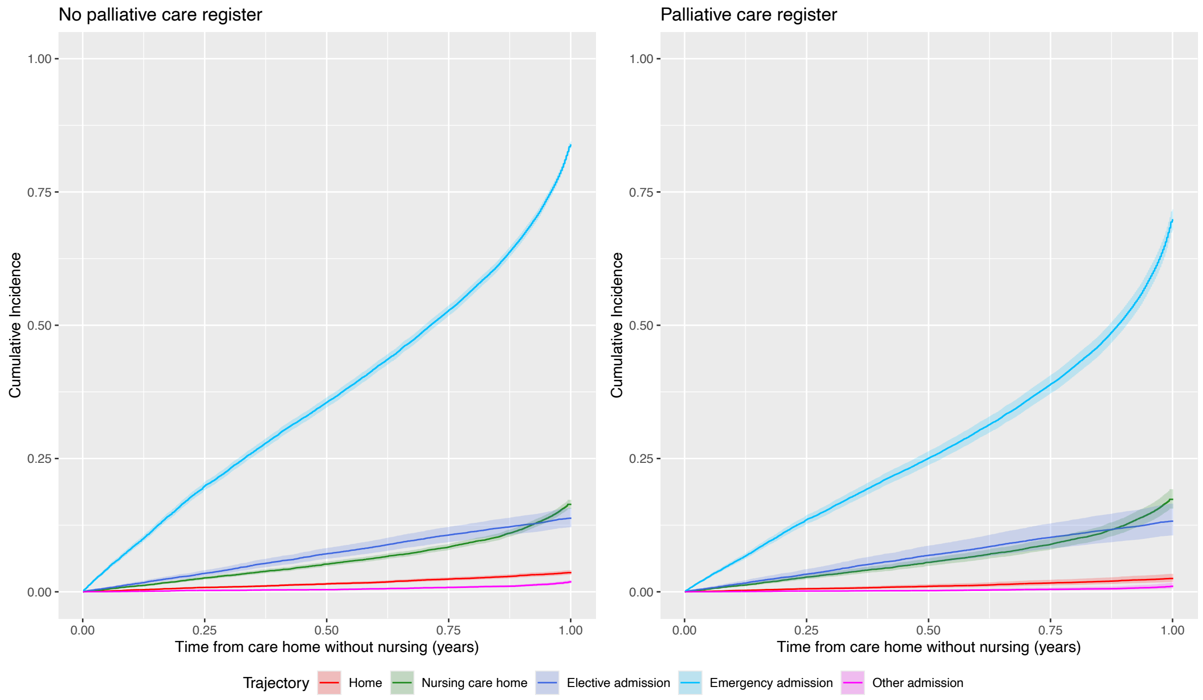

iv) Transitions from emergency hospital admission

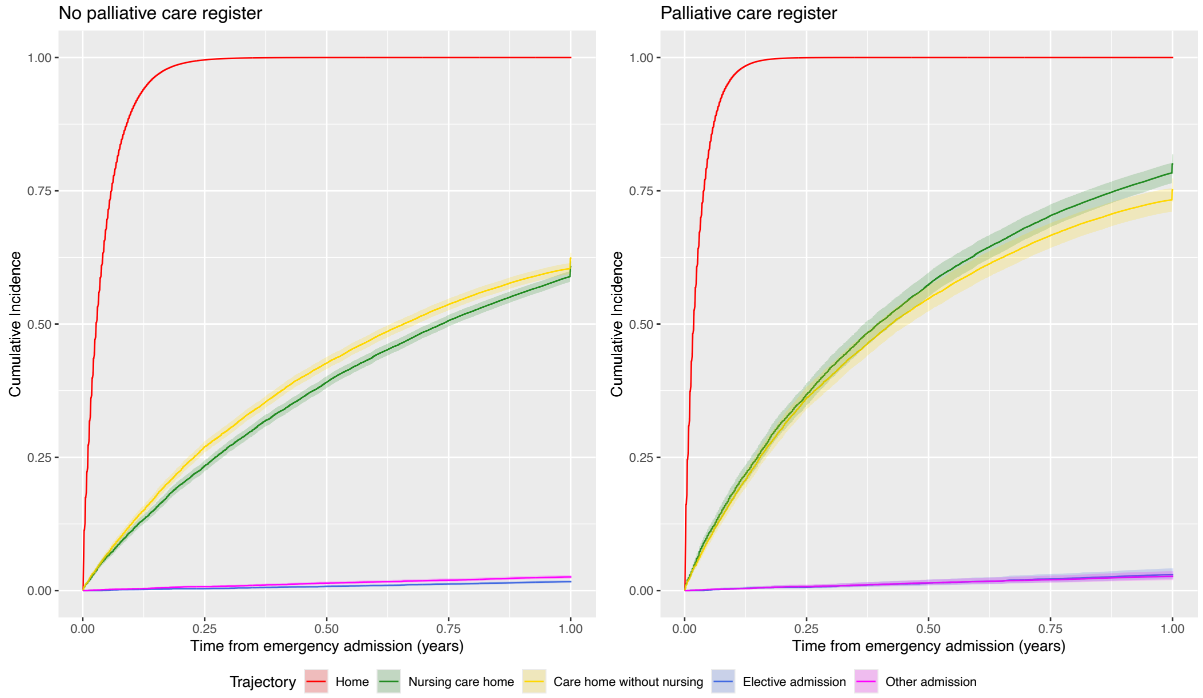

v) Transitions from elective hospital admission

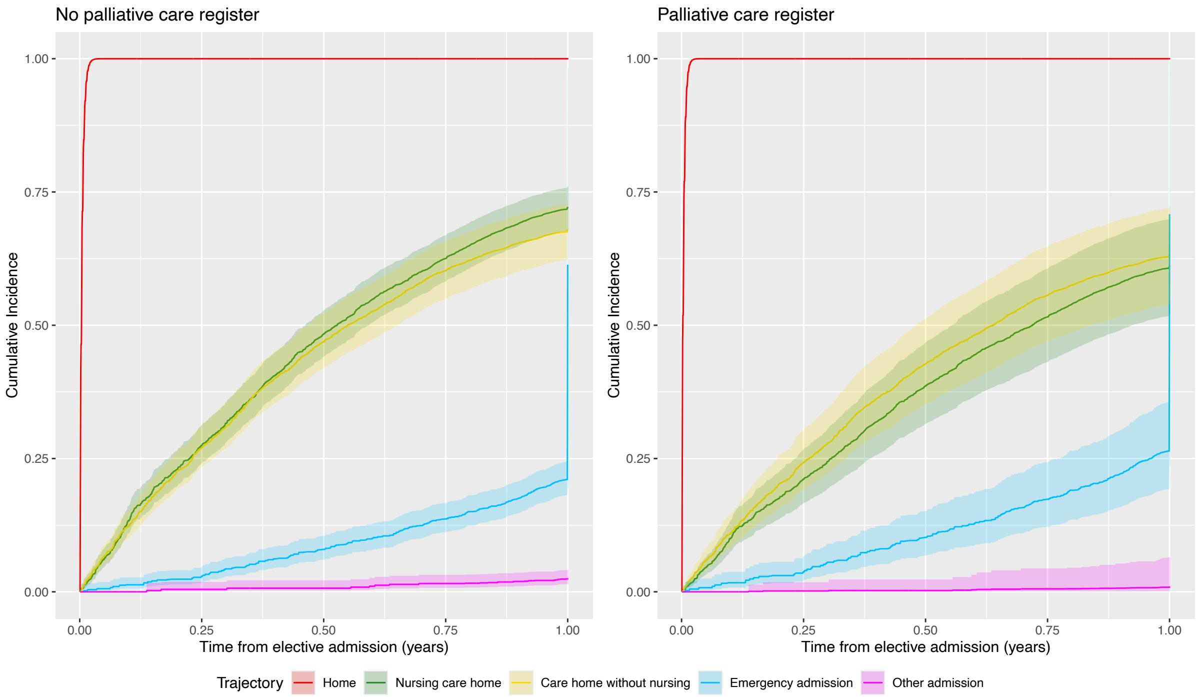

Supplement: Supplementary Tables and Figures [file mmc1.pdf]
